# Chemoenzymatic Preparation and Biophysical Properties of Sulfated Quercetin Metabolites

**DOI:** 10.3390/ijms18112231

**Published:** 2017-10-25

**Authors:** Kateřina Valentová, Kristýna Káňová, Florent Di Meo, Helena Pelantová, Christopher Steven Chambers, Lenka Rydlová, Lucie Petrásková, Alena Křenková, Josef Cvačka, Patrick Trouillas, Vladimír Křen

**Affiliations:** 1 Institute of Microbiology of the Czech Academy of Sciences, Vídeňská 1083, CZ-14220 Prague, Czech Republic; astriik@gmail.com (K.K.); pelantova@biomed.cas.cz (H.P.); christopher.chambers@biomed.cas.cz (C.S.C.); rydlova.l@email.cz (L.R.); petraskova@biomed.cas.cz (L.P.); alenka.petrickova@gmail.com (A.K.); kren@biomed.cas.cz (V.K.); 2 INSERM U850, Univ. Limoges, School of Pharmacy, 2 rue du Docteur Marcland, F-87025 Limoges, France; patrick.trouillas@unilim.fr; 3 Institute of Organic Chemistry and Biochemistry, Czech Academy of Sciences, Flemingovo nám. 2, CZ-16610 Prague, Czech Republic; cvacka@uochb.cas.cz; 4 Regional Centre of Advanced Technologies and Materials, Department of Physical Chemistry, Faculty of Science, Palacký University, tř. 17. listopadu 12, CZ-77146 Olomouc, Czech Republic

**Keywords:** quercetin, sulfotransferase, sulfates, metabolites, antiradical activity, lipid peroxidation, density functional theory, molecular dynamics

## Abstract

Sulfated quercetin derivatives are important authentic standards for metabolic studies. Quercetin-3′-*O*-sulfate, quercetin-4′-*O*-sulfate, and quercetin-3-*O*-sulfate as well as quercetin-di-*O*-sulfate mixture (quercetin-7,3′-di-*O*-sulfate, quercetin-7,4′-di-*O*-sulfate, and quercetin-3′,4′-di-*O*-sulfate) were synthetized by arylsulfotransferase from *Desulfitobacterium hafniense*. Purified monosulfates and disulfates were fully characterized using MS and NMR and tested for their 1,1-diphenyl-2-picrylhydrazyl (DPPH), 2,2′-azinobis-(3-ethylbenzothiazoline-6-sulfonic acid) (ABTS^+^) and *N*,*N-*dimethyl-*p*-phenylenediamine (DMPD) radical scavenging, Folin-Ciocalteau reduction (FCR), ferric reducing antioxidant power (FRAP), and anti-lipoperoxidant activities in rat liver microsomes damaged by *tert*-butylhydroperoxide. Although, as expected, the sulfated metabolites were usually less active than quercetin, they remained still effective antiradical and reducing agents. Quercetin-3′-*O*-sulfate was more efficient than quercetin-4′-*O*-sulfate in DPPH and FCR assays. In contrast, quercetin-4′-*O*-sulfate was the best ferric reductant and lipoperoxidation inhibitor. The capacity to scavenge ABTS^+•^ and DMPD was comparable for all substances, except for disulfates, which were the most efficient. Quantum calculations and molecular dynamics simulations on membrane models supported rationalization of free radical scavenging and lipid peroxidation inhibition. These results clearly showed that individual metabolites of food bioactives can markedly differ in their biological activity. Therefore, a systematic and thorough investigation of all bioavailable metabolites with respect to native compounds is needed when evaluating food health benefits.

## 1. Introduction

Quercetin is a prominent food bioactive flavonol whose daily intake has considerably increased due to its use as food supplements [1] and mainly due to the “eat five fruits and vegetables a day” international recommendation. Unfortunately, quercetin aglycone suffers from low water solubility, poor bioavailability, and instability [2]. Quercetin pharmacokinetics and metabolism have been extensively studied and it was found that after deglycosylation of respective glycosides in the small intestine it is preferentially sulfated, glucuronidated or *O*-methylated by Phase II biotransformation enzymes [3,4]. To date, in most of the studies on bioavailability, the level of quercetin in biological samples (mostly blood plasma and/or urine) was measured as the sum of free and conjugated (after conjugate hydrolysis by gut enzymes from *Helix pomatia*) aglycones). Specific determination of conjugates is typically accomplished using HPLC/MS [2]. The following quercetin conjugates were thus identified in human plasma: quercetin-3-*O*-glucuronide, quercetin glucuronide sulfate (without determination of the conjugation positions), isorhamnetin-3-*O-*glucuronide, quercetin-3′-*O*-sulfate and isorhamnetin [5]. However, the identification of the exact structures of these metabolites (i.e., position of conjugation) requires authentic and well-characterized standards.

Numerous attempts to prepare authentic standards of quercetin metabolites have been published to date. Quercetin-3′-*O*-sulfate, quercetin-7-*O*-sulfate, and quercetin-4′,7-*O*-disulfate were previously prepared by chemical synthesis, which involves a laborious series of protection and deprotection steps, and purification steps with overall low yields and high consumption of reagents [6]. The reaction of quercetin with SO_3_-triethylamine complex without protection steps led to a complex mixture of mono- and disulfates, which was difficult to purify and consequently yields were very low, typically ranging from 0.8% to 16%. After separation by preparative HPLC, the fractions containing monosulfates at C-3 and C-7 in ca 90% purity were obtained; sulfates at C-3′ and C-4′ were not separable. Moreover, the products were characterized by ^1^H NMR only [7]. A chemical synthesis of a whole series of quercetin sulfates, without providing their yields and purities, was recently reported, but the compounds were mostly characterized by LC-MS [3]. On the other hand, alternative methods to produce sulfated metabolites involve chemoenzymatic procedures. The main advantage of these procedures is that the synthesis takes place in mild conditions without the use of expensive, potentially toxic reagents and solvents. Arylsulfotransferase (AST) from *Eubacterium* A-44 was exploited for quercetin sulfation yielding quercetin-3,3′-di-*O*-sulfate and quercetin-3,3′,7-tri-*O*-sulfate without indication of purity or yields [8]. AST from *Desulfitobacterium hafniense* [9,10] preferentially sulfated the catechol moiety of the flavonols [11]; in the case of quercetin, inseparable mixtures of quercetin-3′-*O*-sulfate and quercetin-4′-*O*-sulfate were previously prepared by us [11] and others [10]. The aim of the present study was therefore to prepare pure quercetin sulfates and to evaluate their basic biophysical properties.

## 2. Results and Discussion

### 2.1. Time-Course of Quercetin Sulfation

**Figure 1:**
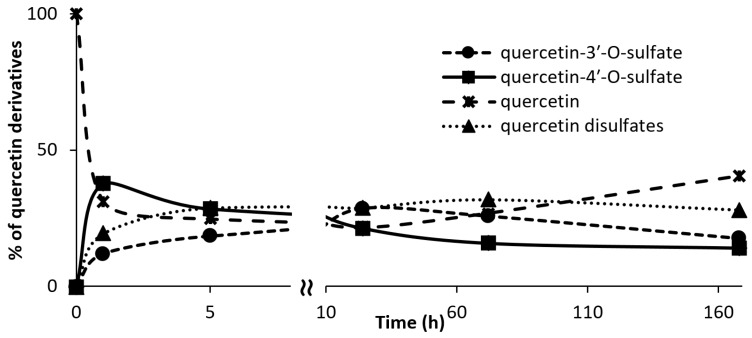
Quercetin sulfation vs. time. The percentage of soluble quercetin derivatives was calculated from the peak areas of the respective compounds. In the case of quercetin disulfates, the total percentage of all isomers was taken into the calculation.

To extend our knowledge about the different quercetin sulfates that can be produced by AST from *D. hafniense* using *p*-nitrophenylsulfate (*p*-NPS) as sulfate donor, the time course of synthesis of all detectable soluble sulfated products was investigated. Their formation, monitored by HPLC, started shortly after addition of the enzyme to the reaction mixture. The concentration of the mono- and disulfates grew except for quercetin-4′-*O*-sulfate, which, after a transient increase within the first hour, gradually decreased. This might be caused by consecutive sulfation yielding disulfates or quercetin-4′-*O*-sulfate hydrolysis. Reaction times longer than ca 5 h were associated with considerable formation of polymeric non-soluble byproducts, but also the hydrolysis of the sulfates, which afforded the starting material quercetin (Figure 1 and Figure S1).

### 2.2. Sulfation by Alternative Sulfate Donors

**Figure 2:**
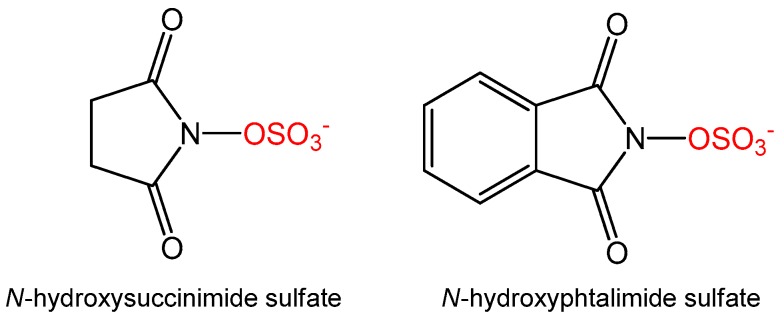
Structures of alternative sulfate donors.

To manipulate the ratio of the sulfates formed we also tested alternative sulfate donors, namely *N*-hydroxysuccinimide sulfate, which was recently reported as a new sulfate donor for sulfation of resveratrol, estradiol, and bisphenol A [12], as well as *N*-phtalimide sulfate (Figure 2), which was specifically designed to improve aromatic interaction with AST or with the aromatic acceptor. The formation of quercetin sulfates was compared with all three sulfate donors, all at 1.2 equivalent. Although the ratio of monosulfates was slightly affected for *N*-hydrosuccimide sulfate, the overall conversion was rather low (67% at 5 h, Table 1) and almost no disulfates were formed. Using *N*-phtalimide sulfate, no sulfates of quercetin were detected at all. We therefore did not follow this approach and we studied in more detail the products of our previously optimized procedure [11].

### 2.3. Purification of the Sulfated Products

As described previously [11], the separation of the sulfated products from the reaction mixture was a highly challenging task. Apart from quercetin mono- and disulfates, the reaction mixture also contained considerable amounts of the parent compound (quercetin, conversion was typically ca 60%), *p*-NPS and *p*-nitrophenol (*p*-NP). Therefore, the mixture had to be pre-purified by extraction with ethyl acetate at pH 7.5–7.7 to remove the bulk of *p*-NP and the aqueous phase was then fractionated on Sephadex LH-20 eluted with 80% MeOH. The formation of two isomeric monosulfates by sulfation of quercetin with AST was previously described [11], but as an inseparable mixture of isomers. After detailed analysis of the fractions from the Sephadex LH-20 chromatography, a slight difference in retention factors of the individual monosulfates was noticed, this enabled their isolation and subsequent purification. To obtain the individual sulfates in sufficient quantity and purity, two to five subsequent chromatographic runs on an LH-20 column were required. During this procedure, all quercetin sulfates purified by gel chromatography exhibited limited solubility in both water and methanol mobile phases. This was presumably caused by removal of the respective counter-ions on LH-20 while the free acids (phenolsulfates) remained insoluble. This problem was resolved by neutralization with aqueous NaOH until dissolution. Particular quercetin monosulfates, i.e., quercetin-3′-*O*-sulfate and quercetin-4′-*O*-sulfate (Figure 2), were prepared at ≥95% purity in overall quantities each over 100 mg.

A fraction containing quercetin disulfates was also repeatedly isolated after quercetin sulfation. However, in ca 50% of cases, the isolated fractions of disulfates decomposed to form quercetin, in agreement with previous studies with chemically prepared sulfated molecules [13]. No monosulfates of other products visible in HPLC were detected in such decomposed samples. Despite numerous attempts, we were not able to identify the exact conditions (pH and temperature during solvent evaporation) that caused this decomposition. For further experiments, only stable disulfate fraction was used and its stability was monitored before each use.

**Figure 3:**
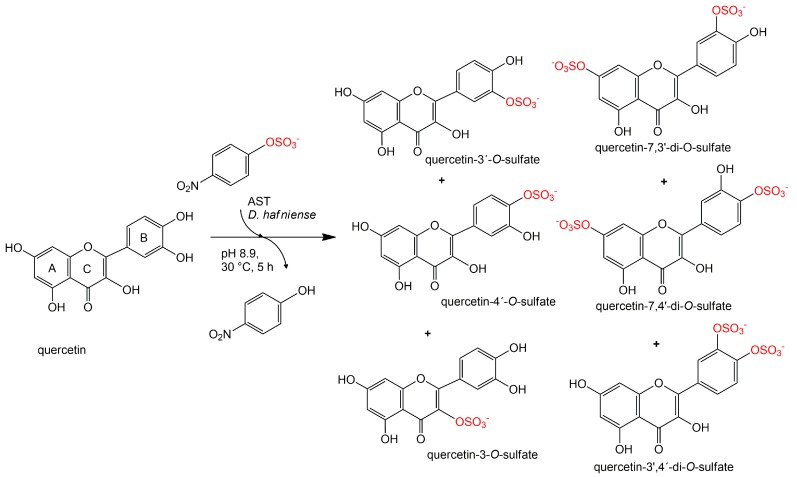
Sulfation of quercetin catalyzed by arylsulfotransferase from *Desulfitobacterium hafniense*.

Formation of a trace product was observed as well in approximately 1% yield. This product was isolated always in a mixture with quercetin-3′,4′-di-*O*-sulfate (ratio ca 4:1) and its structure was determined to correspond to quercetin-3-*O*-sulfate by NMR (Figure 3).

### 2.4. Characterization of the Sulfates

The identity of all monosulfates (namely quercetin-3′-*O*-sulfate, quercetin-4′-*O*-sulfate, and quercetin-3-*O*-sulfate) was confirmed by comparing their ^1^H and ^13^C NMR (Figures S3, S4, S7, S8, S11 and S12) and MS spectra (see Section 3.8. and Figures S5, S9 and S13) with previously published data [3,11,14,15].

Quercetin disulfates were identified in a mixture of three structurally similar compounds; the extracted NMR spectrum of each component (Table 2) showed structural features typical for quercetin. The ^13^C NMR spectrum displayed five aromatic methines and ten quaternary carbons (one C=O). Except for broad hydroxyl singlets, giving no cross-peaks in 2D experiments, the ^1^H NMR spectrum contained two spin systems: AB system of meta-positioned aromatic protons (A-ring) and ABC system of 1,3,4-trisubstituted aromatic ring B. HMBC experiment enabled quaternary carbons to be assigned and joined with the previously mentioned spin systems.

Sulfate attachment at C-7 (A-ring) was indicated by the upfield shift of its signal and downfield shifts of adjacent C-6 and C-8 as described by Barron et al. [6]. The position of the sulfate at catechol moiety (B-ring) was deduced from typical changes of the carbon chemical shifts compared to quercetin: upfield for C-3′ and downfield for C-2′ and C-4′ in 3′-*O*-sulfate, and upfield for C-4′ and downfield for C-3′ and C-5′ in 4′-*O*-sulfate [16]. The structure of quercetin 3′,4′-di-*O*-sulfate was confirmed by the comparison of carbon and proton data (Table 2) of the B-ring with those of previously published 3,7,3′,4′-tetra-*O*-sulfate [17].

Besides NMR data, quercetin-3′-*O*-sulfate and quercetin-4′-*O*-sulfate slightly differed also in their TLC retention factors, HPLC retention times, and UV spectra; for quercetin-3-*O*-sulfate, the differences were more pronounced (Table 3).

The purity of the quercetin-3′-*O*-sulfate and quercetin-4′-*O*-sulfate samples used for determination of radical scavenging activity reached 99% and 97 %, respectively (Table 3, Figures S6 and S10); quercetin-3-*O*-sulfate was not tested due to its paucity. The fraction of disulfates was a mixture composed of the three quercetin-3′,4′-di-*O*-sulfate, quercetin-7,3′-di-*O*-sulfate and quercetin-7,4′-di-*O*-sulfate isomers in a 50:42:8 ratio (Table 3, Figure 3). The overall purity reached 91% (Table 3, Figure S18).

### 2.5. Regioselectivity of Sulfation and Distribution of the Different Isomers

In the present study, we obtained quercetin-3′-*O*-sulfate and quercetin-4′-*O*-sulfate with yields of 15% and 7%, respectively. This agrees with better stability of quercetin-3′-*O*-sulfate compared to quercetin-4′-*O*-sulfate by 1.2 kcal.mol^−1^ (in terms of Gibbs energy, see Table 4). This is mainly attributed to a lower π-conjugation between B- and C-rings (mesomeric effects) in quercetin-4′-*O*-sulfate.

Conversely to the ratio obtained from NMR spectroscopy of the disulfate mixture, the theoretical calculations of stabilizing energy suggested that the mixture should mainly consist of quercetin-7,3′-di-*O*-sulfate and to a lesser extent quercetin-7,4′-di-*O*-sulfate. Indeed, these two disulfates were significantly more stable than quercetin-3′,4′-*O*-disulfate (difference in Gibbs energy between quercetin-3′,4′-*O*-disulfate and both quercetin-7,3′-di-*O*-sulfate and quercetin-7,4′-di-*O*-sulfate of 8.4 and 7.8 kcal.mol^−1^, respectively, see Table 4). This suggests that quercetin-3′,4′-*O*-disulfate is a kinetic product, whereas quercetin-7,3′-di-*O*-sulfate is the major thermodynamic product.

### 2.6. Antioxidant Activity

The reducing capacity of the different quercetin sulfates (except for quercetin-3-*O*-sulfate, which was not tested due to its paucity) towards the Folin-Ciocalteau reagent was determined as gallic acid equivalents (GAE) at pH 10. The reducing capacity was similar for quercetin-3′-*O*-sulfate (1.03 GAE) and quercetin-disulfates (1.01 GAE) but it was higher for quercetin-4′-*O*-sulfate (1.52 GAE) and quercetin (1.72 GAE). As expected, all tested sulfates were less active than quercetin (IC_50_ 3.4 μM) as 1,1-diphenyl-2-picrylhydrazyl (DPPH) free radical scavengers. Namely, quercetin-3′-*O*-sulfate (IC_50_ 6.26 μM) exhibited approximately half of the quercetin activity, whereas quercetin-4′-*O*-sulfate was substantially less active (IC_50_ 23.0 μM). Interestingly, quercetin-di-*O-*sulfates (IC_50_ 7.35 μM) were as active as quercetin-3′-*O*-sulfate. The antioxidant capacity in aqueous milieu was evaluated as the capacity to scavenge 2,2′-azinobis-(3-ethylbenzothiazoline-6-sulfonic acid) (ABTS^+^) radical and the activities were given as trolox equivalents (TE). The mixture of quercetin-di-*O-*sulfates (1.44 TE) was slightly less active than quercetin-3′-*O*-sulfate, quercetin-4′-*O*-sulfate, and quercetin, while all three exhibited similar activities (≅1.9 TE). *N*,*N-*Dimethyl-*p*-phenylenediamine (DMPD) radical scavenging activity of quercetin and its monosulfates was comparable (≅1.0 vitamin C equivalents, CE), the disulfates were significantly more active (1.6 CE). Ferric reducing antioxidant power (FRAP) of quercetin was surprisingly more pronounced for all sulfates (>1.3 CE) compared with quercetin (0.8 CE); quercetin-4′-*O*-sulfate was the most active (2.3 CE). Finally, the capacity of the sulfates to inhibit lipoperoxidation induced by *t*-butyl hydroperoxide (*t*BH) in rat liver microsomes showed that quercetin-4′-*O*-sulfate was the most active (IC_50_ 9.32 μM), e.g., ca twice more active than quercetin (19.84 μM). A lower activity was observed for quercetin-3′-*O*-sulfate (IC_50_ 34.25 μM) and the disulfates were the least active (IC_50_ 48.32 μM, Table 5).

Biological activity of quercetin sulfates has only been studied to a limited extent to date, with the exception of ABTS^+•^ scavenging and FRAP [18]. Published data agree with our results for quercetin monosulfates, but differ for quercetin, which had significantly higher activity; the disulfates were not studied in this work [18]. The discrepancy in the case of quercetin is attributed to methodological differences (e.g., use of myoglobin vs. persulfate for the induction of radical formation). In contrast, the results of individual tests for quercetin agree with our previously published data [19]. On the other hand, antioxidant activity (ABTS^+•^, DPPH and superoxide radical scavenging) of a mixture of 12 quercetin metabolites from pig urine was significantly stronger compared to quercetin [20].

### 2.7. Theoretical Rationalization of Free Radical Scavenging of Quercetin Sulfates

This study has highlighted differences in activity between isomeric quercetin monosulfates (Table 4). In the case of DPPH radical scavenging, quercetin-3′-*O*-sulfate was ca 4× more active than the quercetin-4′-*O*-sulfate. The same trend was observed in the case of FCR. The capacity to scavenge ABTS^+•^ radical was comparable for all compounds (1.9 TE) except for the disulfate fraction (1.4 TE). Density Functional Theory (DFT) calculations were performed to support rationalization of free radical scavenging capacities.

It has repeatedly been shown that the OH groups of C- and B-rings of flavonoids play a crucial role in free radical scavenging capacity, by H atom transfer mechanism [21,22]. This capacity can be efficiently evaluated by O-H bond dissociation enthalpy (BDE); the lower the BDE, the higher the H atom transfer capacity. The sulfate moieties do not significantly influence the 3-OH BDEs of all derivatives lying within the same range (ca 78 kcal.mol^−1^, see Table 4). The 3-OH group thus provides an equal contribution to free radical scavenging activities. Likewise, the electron transfer capacity was not modified by the presence of the sulfate moieties, ionization potentials (IPs) being ca 6.1 eV (Table 4). Therefore, the differences observed between sulfate derivatives in free radical scavenging capacity (mainly DPPH scavenging) are attributed almost exclusively to the B-ring. Indeed, the 3′- and 4′-OH groups exhibited higher BDEs (lower H-atom donor capacity) in the presence of an adjacent sulfate moiety, owing to the relatively strong intramolecular H-bond, which was stronger in quercetin sulfates with respect to quercetin itself. The BDEs remained however sufficiently low to favor efficient free radical scavenging (Table 4). When the B-ring is monosulfated, one active group is lost. As expected, when blocking the 4′-OH group (quercetin-4′-*O*-sulfate), the most active group is lost, only 3′-OH remains active in the B-ring with a BDE of 83.4 kcal.mol^−1^ (Table 4). Besides, when blocking the 3′-OH group (quercetin-3′-*O*-sulfate), the 4′-OH remains with a lower BDE of 81.3 kcal.mol^−1^ (Table 4). This fully agrees with the higher antioxidant activity of quercetin-3′-*O*-sulfate compared to quercetin-4′-*O*-sulfate, however the activity was a half compared to quercetin (Table 4).

In the quercetin-di-*O*-sulfate mixture, the presence of quercetin-3′,4′-di-*O*-sulfate (having no DPPH scavenging activity) is likely to dramatically decrease the net activity. As the activity of the disulfate mixture is only slightly lower than that of quercetin-3′-*O*-sulfate, one can speculate that not only quercetin-7,3′-di-*O*-sulfate participates in the free radical scavenging capacity. The presence of (i) a small amount of the active quercetin-7,3′-di-*O*-sulfate, and (ii) the highly active quercetin-3-*O*-sulfate, found as a trace in the mixture, most probably compensates for the decrease in DPPH scavenging activity attributed to quercetin-3′,4′-di-*O*-sulfate in the disulfate mixture.

### 2.8. Theoretical Rationalization of Lipid Bilayer Insertion and Lipoperoxidation Inhibition

**Figure 4:**
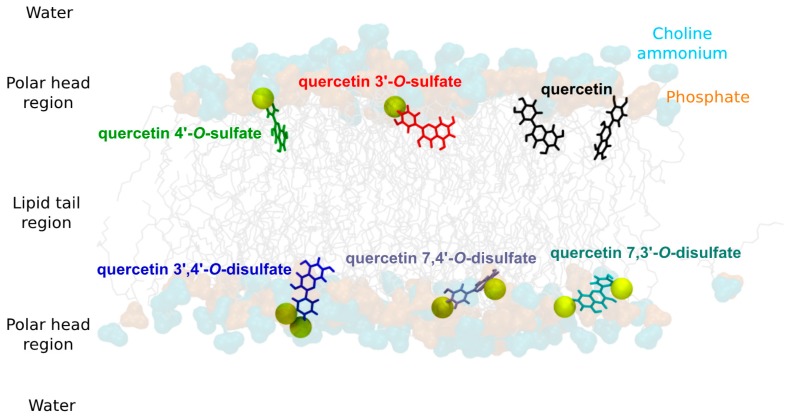
Representative snapshots of quercetin derivatives in 1-palmitoyl-2-oleoyl-*sn*-glycero-3-phosphocholine (POPC) membrane. Phosphate and choline ammonium moieties are depicted in orange and cyan, respectively. Sulfate moieties of each solute are pictured in yellow van der Waals radii.

Surprisingly quercetin-4′-*O*-sulfate was the most efficient lipid-peroxidation inhibitor, ca 4× more active than the 3′-*O*-sulfate (IC_50_ of 9 and 34 μM, respectively). The efficiency of quercetin derivatives as lipid peroxidation inhibitors does not only depend on their intrinsic free radical scavenging capacity but also on their partitioning in lipid bilayer membrane, mainly focusing on depth of insertion. Molecular dynamics (MD) simulations were performed in a 1-palmitoyl-2-oleoyl-*sn*-glycero-3-phosphocholine (POPC) bilayer for quercetin and the different sulfates synthesized in this study. As already described [23], quercetin inserts and partitions in lipid bilayer, and it is located under the high density polar head region, at 12.5 ± 2.0 Å (Table 6). The presence of the sulfate moieties significantly affects partitioning. The negative charge of the sulfates indeed acts as an anchor to lipid polar head groups by substantially increasing electrostatic noncovalent interactions with choline ammonium moieties of phospholipids, i.e., E_elec_ increased (in absolute value) with the degree of sulfation (ca 50 kcal.mol^−1^, ca 90 kcal.mol^−1^ and more than 150 kcal.mol^−1^ for quercetin, mono sulfates and disulfates, respectively, see Table S1 and Figure S2). As a result, the depth of insertion is as follows: quercetin > quercetin-4′-*O*-sulfate > quercetin-3′-*O*-sulfate > quercetin-7,4′-di-*O*-sulfate ≥ quercetin-7,3′-di-*O*-sulfate > quercetin-3,4′-di-*O*-sulfate (see Table 6 and Figure 4). The importance of electrostatic interactions with polar head region is particularly crucial with quercetin-3′,4′-di-*O*-sulfate, rationalizing its shallower insertion with respect to the other two other disulfates (E_elec_ = −245.5, −170.1, and −163.1 kcal.mol^−1^ for quercetin-3′,4′-di-*O*-sulfate, quercetin-7,3′-di-*O*-sulfate, and quercetin-7,4′-di-*O*-sulfate, respectively).

**Figure 5:**
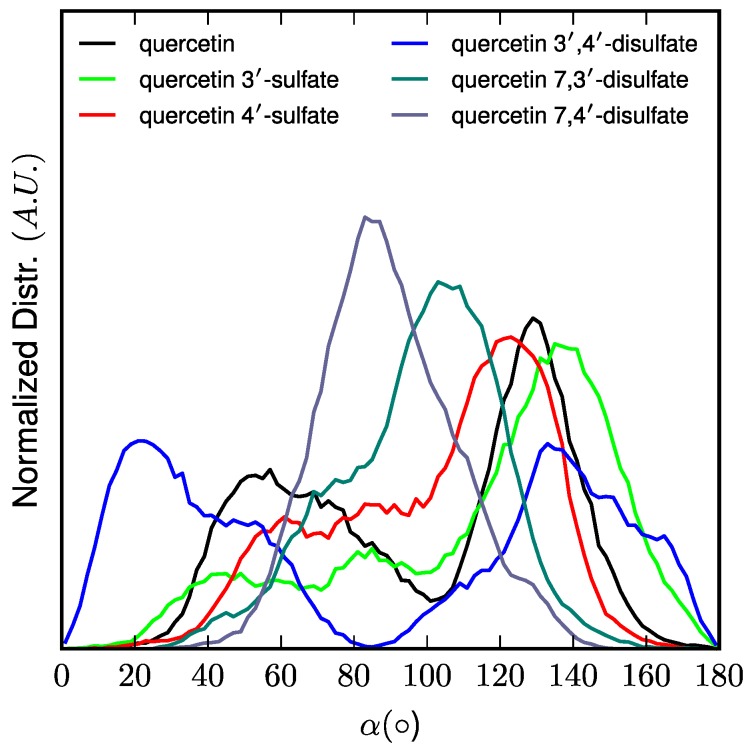
Normalized distribution of the orientation of quercetin derivatives with respect to membrane normal axis (z) pictured by the α-angle.

Interestingly, the location of the C-ring is strongly correlated to the orientation (followed by the α-angle, see Figure 5) of quercetin derivatives. The longest axis of quercetin-3′,4′-di-*O*-sulfate is parallel to the normal to the membrane surface (z-axis), the B-ring being strongly anchored to the lipid polar head region owing to the two sulfate moieties. In contrast, sulfate moieties of quercetin-7,3′-di-*O*-sulfate and quercetin-7,4′-di-*O*-sulfate are on the A- and B-rings maintaining the overall orientation perpendicular to the *z*-axis. Finally, B-rings of quercetin, quercetin-3′-*O*-sulfate, and quercetin-4′-*O*-sulfate mainly bind to the polar head region. However, such binding allows more flexibility than with disulfates, leading to a wider distribution of α-angle around 90° (Figure 5).

**Figure 6:**
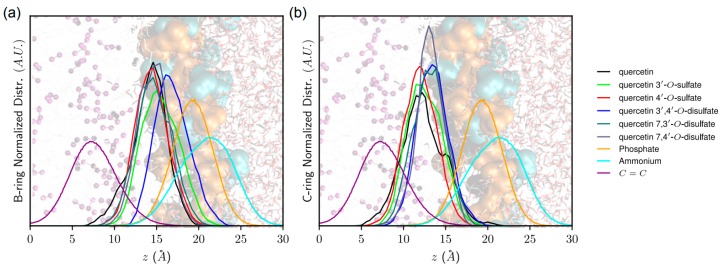
Normalized distribution of (**a**) B-ring and (**b**) C-ring of quercetin derivatives with respect to membrane normal axis (z). Background picture represents water-lipid bilayer interface, highlighting (from left to right) water phase, polar head charged moieties (choline and phosphate moieties depicted in blue and orange, respectively) as well as lipid leaflet region in which POPC C=C double bonds are colored in purple.

Particular attention was paid to the free-radical-scavenging active groups [20,24], namely 3′-OH, 4′-OH, and 3-OH (see Table 6 and Figure 6), and their depth of insertion. The closer the distance to lipid unsaturation (where peroxy radicals are formed), the higher is the capacity to inhibit the propagation stage of lipid peroxidation. Sulfate moieties pull the compounds far from the middle of the lipid bilayer and lipid unsaturation; however, this greatly depends on the position of the substitution. Interestingly, quercetin-4′-*O*-sulfate exhibits slightly deeper B-ring partitioning with respect to quercetin-3′-*O*-sulfate. This is confirmed by a greater interaction energy between quercetin-4′-*O*-sulfate and lipid tails, than with quercetin-3′-*O*-sulfate (E_int_ = −23.0 and −29.4 kcal.mol^−1^, for quercetin-3′-*O*-sulfate and quercetin-4′-*O*-sulfate, respectively). Moreover, the (antioxidant active) 3-OH group of quercetin-4′-*O*-sulfate lies deeper in the bilayer, so it is more prone to efficiently inhibit the propagation stage of lipid peroxidation. It must be stressed that MD simulations were performed in the non-oxidized POPC bilayer. Hydroperoxide groups formed from the oxidation of lipid C=C double bonds are known to (i) be located closer to the polar head region than C=C bond and (ii) increase water permeation in lipid bilayer membranes [25]. Therefore, overlap between the 3-OH group and lipid hydroperoxide region is expected to be enhanced with quercetin-4′-*O*-sulfate.

## 3. Materials and Methods

### 3.1. Chemicals and Reagents

Quercetin hydrate, 1,1-diphenyl-2-picrylhydrazyl (DPPH) radical, antioxidant assay kit (CS0790), pooled microsomes from male rat liver (M9066), dimethyl sulfoxide (DMSO), *p*-NPS, tert-butyl hydroperoxide, trolox, and other chemicals were obtained from Sigma-Aldrich (Prague, Czech Republic). Folin-Ciocalteau reagent was purchased from Merck (Prague, Czech Republic), DMPD and FRAP kits from Bioquochem (Llanera, Spain).

### 3.2. NMR Spectroscopy

NMR spectra were recorded on a Bruker Avance III 600 MHz spectrometer (600.23 MHz for ^1^H, 150.94 MHz for ^13^C) at 30 °C in DMSO-*d*_6_. Residual signal of solvent (δ_H_ 2.500 ppm, δ_C_ 39.60 ppm) was used as an internal standard. NMR experiments ^1^H NMR, ^13^C NMR, gCOSY, gHSQC, and gHMBC were performed using the manufacturer’s software (Topspin 3.2, Bruker BioSpin, Rheinstetten, Germany). ^1^H NMR and ^13^C NMR spectra were zero filled to fourfold data points and multiplied by window function before Fourier transformation. Two-parameter double-exponential Lorentz-Gauss function was applied for ^1^H to improve resolution and line broadening (1 Hz) was applied to get better ^13^C signal-to-noise ratio. Chemical shifts are given in δ-scale with digital resolution justifying the reported values to three (δ_H_) or two (δ_C_) decimal places, respectively.

### 3.3. Mass Spectrometry

Mass spectra in the negative ion mode were measured using a LTQ Orbitrap XL hybrid mass spectrometer (ThermoFisher Scientific, Waltham, MA, USA) equipped with an electrospray ion source. The samples were dissolved in methanol and introduced into the mobile phase flow (methanol/water 4:1; 100 μL/min) using a 2-μL loop. Spray voltage, capillary voltage, tube lens voltage and capillary temperature were 4.0 kV, −16 V, −120 V, and 275 °C, respectively.

### 3.4. Analytical HPLC–PDA

All analytical HPLC analyses were performed with the Shimadzu Prominence System (Shimadzu, Kyoto, Japan) consisting of a DGU-20A mobile phase degasser, two LC-20AD solvent delivery units, a SIL-20AC cooling auto sampler, a CTO-10AS column oven, and a SPD-M20A diode array detector. Chromatographic data were collected and processed using Shimadzu LabSolutions software (version 5.75 SP2, Shimadzu Corporation, Tokyo, Japan) at a 40 Hz rate.

The separation of quercetin sulfates was achieved on the core-shell silica column Kinetex 5 μm PFP (pentafluorophenyl), 150 mm × 4.6 mm (Phenomenex, CA, USA), thermostated at 40 °C and equipped with a guard column (Analytical Guard Cartridge System, Security guard cartridges (PFP 4 × 3.0 mm ID), Phenomenex)) using linear gradient: water/trifluoroacetic acid (100/0.1, *v*/*v*, phase A) and methanol (phase B); 0–25 min 40–80% B; flow rate 0.6 mL/min. The PDA data were acquired in the 200–450 nm range and the wavelength 360 nm signal was extracted.

### 3.5. Synthesis of Alternative Sulfate Donors

*N*-Hydroxysuccinimide (115 mg, 1 mmol, 1 eq) was dissolved in dioxane (5 mL) and NaH, in a 60% oil dispersion (44 mg, 1.1 mmol, 1.1 eq), was added. The whole was stirred at room temperature for 1 h before slow addition of sulfur trioxide triethylamine complex (199 mg, 1.1 mmol, 1.1 eq). The mixture was stirred overnight until TLC analysis (9:1 EtOAc:MeOH) showed full consumption of starting material. The reaction was quenched with water and the aqueous layer was washed with EtOAc (2 × 10 mL) and concentrated in vacuo. The solid was washed in hot MeOH (2 × 5 mL) to yield *N*-succinimide sulfate as a white solid (109 mg, 56%, ESI–*m*/*z* 194 [M − H]^+^).

*N*-Hydroxyphthalimide (326 mg, 2 mmol, 1 eq) was dissolved in dioxane (5 mL) and to this was added NaH in a 60% oil dispersion (88 mg, 2.2 mmol, 1.1 eq). The reaction mixture was stirred at room temperature for 1 hour before slow addition of sulfur trioxide triethylamine complex (398 mg, 2.2 mmol, 1.1 eq). The reaction was kept at room temperature overnight and TLC (95:5 EtOAc:MeOH) analysis showed full consumption of starting material. The reaction was quenched with water and the aqueous layer was washed with EtOAc (2 × 10 mL) and concentrated in vacuo. The solid was washed in hot MeOH (2 × 5 mL) to yield *N*-phthalimide sulfate as a white solid (79 mg, 17%, ESI–(*m*/*z*) 242 [M − H]^+^). BaCl_2_ test was used to control the presence of both sulfates during the purification procedures.

### 3.6. AST from Desulfitobacterium Hafninense

The expression of the AST enzyme was performed as described by van der Horst et al. [24] with the modifications described in our previous work [11].

### 3.7. Kinetics of Regioisomer Quercetin Sulfate Formation

Quercetin (100 mg, 0.332 mmol, Sigma-Aldrich) was dissolved in 2.5 mL of acetone. *p*-Nitrophenyl sulfate (*p-*NPS, Sigma-Aldrich, 102 mg, 0.399 mmol, 1.2 eq.), AST from *D. hafniense* (360 U·mL^−1^ of the reaction mixture) and Tris-glycine buffer (to the final volume of 18 mL, 100 mM, pH 8.9) were added to the substrate solution and the mixture was incubated at 30 °C under argon atmosphere. The reaction progress was monitored by HPLC. The reaction was stopped after 1, 4, 24, 72, 168, and 336 h.

### 3.8. Quercetin Sulfation Using Alternative Sulfate Donors

Quercetin (50 mg, 0.166 mmol, Sigma-Aldrich) was dissolved in 1 mL of acetone. *p-*NPS (51 mg, 0.199 mmol 1.2 eq.), *N*-succinimide sulfate (38.6 mg, 0.199 mmol 1.2 eq.) or *N*-phthalimide sulfate (48.4 mg, 0.199 mmol 1.2 eq.), AST from *D. hafniense* (360 U·mL^−1^ of the reaction mixture) and Tris-glycine buffer (to a final volume of 9 mL, 100 mM, pH 8.9) was added to the substrate solution and the mixture was incubated at 30 °C under argon atmosphere. The reaction progress was monitored by HPLC. The reaction was stopped after 1 and 5 h.

### 3.9. Preparation and Purification of Quercetin Sulfates

Quercetin (200 mg, 0.664 mmol, Sigma-Aldrich) was dissolved in 5 mL of acetone. 24 mL of 100 mM Tris-glycine buffer (pH 8.9), *p*-NPS (205 mg, 0.80 mmol) and AST from *D. hafniense* (2 mL, 360 U/mL of the reaction mixture) were added to the substrate solution and the mixture was incubated ca 5 h at 30 °C under argon atmosphere. The reaction progress was monitored by HPLC or by TLC (ethyl acetate/methanol/HCO_2_H, 4:1:0.01). The reaction mixture was halved by evaporation in vacuo so that all organic solvents were removed, pH was adjusted to 7.5–7.7 and *p*-NP and residual starting materials were removed by extraction (3 × 50 mL EtOAc). The aqueous phase (15 mL) containing the sulfated products was evaporated, the residue was dissolved in 2 mL of 80% methanol and loaded onto a Sephadex LH-20 (GE Healthcare Bio-Sciences, Uppsala, Sweden) column (30 g dry weight, 3 cm i.d.) packed and equilibrated with 80% aqueous methanol. The elution typically took 2–4 days. The fractions were analyzed by TLC (EtOAc/MeOH/HCO_2_H, 4:1:0.01, *v*/*v*) and the fractions containing the respective product were collected and evaporated in vacuo at 45 °C.

Quercetin-3′-*O*-sulfate was obtained as a yellowish solid (37 mg, yield 15%, purity 99%, Figure S6), its structure was confirmed by ^13^C and ^1^H NMR (Figures S3 and S4) whose results agreed well with published data [11]; HRMS (*m*/*z* calcd for [M−H]^−^ (C_15_H_9_O_10_S) 380.99219, found 380.99158; Figure S5).

Quercetin-4′-*O*-sulfate was obtained as a yellowish solid (18 mg, yield 7%, purity 97%, Figure S10), its structure was confirmed by ^13^C and ^1^H NMR (Figures S7 and S8) whose results agreed well with published data [11]; HRMS (*m*/*z* calcd for [M−H]^−^ (C_15_H_9_O_10_S) 380.99219, found 380.99149; Figure S9).

Quercetin-3-*O*-sulfate was obtained as a yellowish solid (3 mg, yield 1%, purity 80%, Figure S14), its structure was confirmed by ^13^C and ^1^H NMR (Figures S11 and S12) whose results agreed well with published data [14,15]; HRMS (*m*/*z* calcd for [M−H]^−^ (C_15_H_9_O_10_S) 380.99219, found 380.99150; Figure S13).

Quercetin-di-*O*-sulfates mixture was obtained as a yellowish solid (34 mg, yield 10%, purity 91%, Figure S18), its composition was determined by ^13^C and ^1^H NMR. Complete assignment of all extracted NMR signals was accomplished by the use of a combination of gCOSY, gHSQC, and gHMBC experiments (Table 2, Figures S15 and S16); HRMS (*m*/*z* calcd for [M − 2H + Na]^−^ (C_15_H_8_O_13_NaS_2_) 482.93095, found 482.93064; calcd for [M − 2H + K]^−^ (C_15_H_8_O_13_KS_2_) 498.90489, found 498.90447; Figure S17).

### 3.10. Antioxidant Activity Measurement

Reducing capacity was evaluated using Folin–Ciocalteau reagent [26] with minor modifications as described previously [27,28,29]. Antiradical activity was evaluated spectrophotometrically as the ability of the substances to reduce the DPPH radical as described previously [30] with minor modifications [27,28,29]. DMPD [31] radical scavenging and FRAP [32] were measured using kits from Bioquochem (Llanera–Asturias, Spain). The capacity to scavenge the radical cation (ABTS^+•^) was evaluated using the Antioxidant Assay Kit (CS0790, Sigma-Aldrich) and expressed as trolox equivalents (TE) from the trolox calibration curve. Inhibition of microsomal lipid peroxidation was tested using pooled microsomes from male rat livers oxidatively damaged by *tert*-butylhydroperoxide in PBS. Determination of lipid peroxidation products as thiobarbituric acid reactive substances (TBARS) and calculation of the IC_50_ values were performed as previously described [27,28,29].

### 3.11. Statistical Analysis

All data were analyzed with one-way ANOVA, Scheffé and Least Square Difference tests for post hoc comparisons among pairs of means using the statistical package Statext ver. 2.1 (Statext LLC, Wayne, NJ, USA). Differences were considered statistically significant when *p* < 0.05.

### 3.12. Molecular Modeling

For MD simulations, the force field (FF) parameters of the quercetin sulfates were derived from the Generalized Amber Force Field (GAFF) [33] using the antechamber package [34]. Partial atomic charges were derived from RESP (Restrained fit of ElectroStatic Potential) based on calculations achieved within the density functional theory (DFT) formalism with the (IEFPCM)-B3LYP/cc-pVDZ method, in diethylether [35]. The DFT calculations and the atomic charge fitting were performed with the Gaussian09 RevA [36] and RESP-v.III softwares [37], respectively. The three quercetin disulfate derivatives were considered, namely quercetin 7,4′-, 7,3′-, and 3′,4′-di-*O*-sulfates. The lipid14 FF [38] available in the Amber16 package [39] was used to describe POPC lipids. The “three-point” TIP3P water model [40] was used to describe water molecules.

Pure POPC bilayer membranes made of 128 lipids were created using the membrane bilayer builder from the CHARMM-GUI server [41]. Membranes were solvated with a hydration number of 50 water molecules per one lipid molecule. Na^+^ and Cl^−^ ions were added to match with experimental conditions (i.e., (NaCl) = 0.154 M). MD simulations were carried out using both the CPU and GPU codes available in Amber16 [39,42]. Particle-Mesh Ewald (PME) MD simulations were first run on the pure POPC bilayer membrane which was carefully prepared as follows: minimization of the water molecule system prior to the entire system minimization; slow thermalization of water molecules up to 100 K in the (*N*, *V*, *T*) ensemble for 200 ps; thermalization of the whole system to the final temperature (298.15 K) of the entire system for 500 ps (*N*, *P*, *T*); equilibration of the density of the system for 5 ns (*N*, *P*, *T*) MD simulations; finally, production of 200 ns MD simulation. Quercetin sulfates were then inserted into the equilibrated membranes, and the system was relaxed by a short minimization, so as to prevent any steric clash artifact. For each system of interest, four non-interacting identical quercetin sulfates were included in the MD box to fasten sampling. For each system, 400 ns MD simulations were then carried out. The total MD simulation time for the six systems (quercetin, quercetin-3′-*O*-sulfate, 4′-*O*-sulfate, and quercetin-7,4′-, 7,3′-, and 3′,4′-di-*O*-sulfates) was ca 2.4 μs. The analyses were performed (i) over the last 200 ns of the MD trajectories (series of snapshots of the molecular systems) and (ii) considering each non-interacting quercetin separately in the MD box. This allowed a complete sampling of structural properties during 4 × 200 ns to be obtained, after the equilibrium was reached (i.e., within the first 200 ns of the MD simulation). PME MD simulations were carried out using the SHAKE algorithm and a 10 Å noncovalent interaction cut-off. The temperature was maintained using the Langevin dynamics with a collision frequency of 1 ps^−1^. Anisotropic pressure scaling was used in which the pressure relaxation time was set at 1 ps. The analyses were carried out using the cpptraj software [43]. The z-axis is defined as being perpendicular to the membrane surface. The distance of quercetin derivatives to the membrane center was measured as the *z*-component of the vector originating at the center-of-mass of the lipid bilayer and pointing towards the quercetin derivative center-of-mass. The orientation of quercetin and quercetin sulfates in the lipid bilayer membrane was assessed as the α-angle between the *z*-axis and the vector starting from the A- to the B-ring. Noncovalent interactions energies were obtained from MD simulations by calculating the averaged electrostatic and van der Waals interaction energies between (i) quercetin derivatives and POPC molecules, as well as (ii) quercetin and lipid tails only, lipid tails being defined as the *sn*_1_- and *sn*_2_-chains. It is worth noting that such interaction energies can be compared only in systems containing the same number of atoms (i.e., within monosulfate or disulfate derivatives).

Antioxidant properties in the water of quercetin derivatives as well as relative stabilities were assessed at the (DFT) B3P86/cc-pVDZ level of theory at 298 K. Solvent effects were taken into account during optimization by using the integral equation formalism polarizable continuum model (IEFPCM). Ground state geometries were confirmed by a vibrational frequency analysis that indicated the absence of imaginary frequency. Electron transfer capacity was assessed by calculating ionization potentials (IP) as follows IP = E_ArOH_ (ArOH^+•^)–E_ArOH_ (ArOH) where E_ArOH_ pictures energy calculated using the optimized ArOH geometry. H-atom transfer capacities were assessed by calculating ArO-H bond dissociation enthalpies as follows: BDE(ArO-H) = *H*[ArO^•^, 298 K] + *H*[H^•^, 298 K] – *H*[ArOH, 298 K,] where *H* is enthalpy.

## 4. Conclusions

Using arylsulfotransferase from *Desulfitobacterium hafniense* we synthetized and fully characterized a series of potential quercetin sulfated metabolites, namely quercetin-3′-*O*-sulfate, quercetin-4′-*O*-sulfate, and quercetin-3-*O*-sulfate as well as a quercetin-di-*O*-sulfate mixture (quercetin-7,3′-di-*O*-sulfate, quercetin-7,4′-di-*O*-sulfate, and quercetin-3′,4′-di-*O*-sulfate). The quercetin sulfated metabolites are usually less active as radical scavengers, and reducing or anti-lipoperoxidant agents than quercetin itself. However, they appeared still effective antiradical and reducing agents. While quercetin-3′-*O*-sulfate was more efficient than quercetin-4′-*O*-sulfate in DPPH and FCR assays, quercetin-4′-*O*-sulfate was the best ferric reductant and lipoperoxidation inhibitor. The capacity to scavenge ABTS^+•^ and DMPD was comparable for all substances, except for disulfates, which were the most efficient. Quantum calculations and molecular dynamics simulations on membrane models supported rationalization of free radical scavenging and lipid peroxidation inhibition. These results clearly showed that individual metabolites of the food bioactives can markedly differ in their biological activity. Therefore, a systematic and thorough investigation of all bioavailable metabolites with respect to native compounds is always required when evaluating food health benefits.

## Figures and Tables

**Table 1:** Comparison of quercetin sulfation using alternative sulfate donors.

Sulfate Donor	*p*-Nitrophenyl Sulfate	*N*-Hydroxysuccinimide Sulfate	*N*-Hydroxyphtalimide Sulfate
Quercetin 3′-*O*-sulfate	57%	67%	0%
Quercetin 4′-*O*-sulfate	33%	30%	0%
Quercetin di-*O*-sulfates	8%	2%	0%
Conversion	79%	67%	0%

Results are expressed as percentage of soluble quercetin derivatives, calculated from the peak areas of the respective compounds. In the case of quercetin disulfates, the total percentage of all isomers was taken into the calculation. All donors were used at 1.2 eq.

**Table 2:** ^13^C and ^1^H NMR data of quercetin-3′,4′-di-*O*-sulfate, quercetin-7,3′-di-*O*-sulfate, and quercetin-7,4′-di-*O*-sulfate (600.23 MHz for ^1^H, 150.93 MHz for ^13^C, DMSO-*d*_6_, 30 °C).

Sulfate Position	3′,4′	7,3′	7,4′		3′,4′	7,3′	7,4′		
Atom	δ_C_ (ppm)			m	δ_H_ (ppm)			m	*J* (Hz)
2	145.96	146.94	146.66	s	-	-	-	-	-
3	136.43	136.43	n.e.	s	-	-	-	-	-
4	176.05	176.29	176.5 ^1^	s	-	-	-	-	-
5	160.82	159.82	159.83	s	-	-	-	-	-
6	98.35	101.38	101.33	d	6.179	6.570	6.553	d	2.0
7	164.23	159.39	159.50	s	-	-	-	-	-
8	93.40	97.53	97.51	d	6.392	6.949	6.988	d	2.0
9	156.25	155.21	155.32	s	-	-	-	-	-
10	103.10	105.07	105.13	s	-	-	-	-	-
1′	124.48	122.20	125.16	s	-	-	-	-	-
2′	119.85	123.16	116.17	d	8.321	8.108	7.71 ^2^	d	2.3
3′	143.47	140.81	148.52	s	-	-	-	-	-
4′	146.28	151.47	142.92	s	-	-	-	-	-
5′	119.28	117.34	121.95	d	7.684	6.990	7.404	d	8.6
6′	122.55	124.88	119.33	d	7.773	7.846	7.629	dd	2.3, 8.6

m: multiplicity; n.e: not extracted; ^1^ broad signal; ^2^ HSQC readout.

**Table 3:** Quercetin sulfates obtained using arylsulfatase: analytical characteristics.

Compound	Purity (%) ^a^	Retention Time (min) ^a^	R_f_^b^	UV ^c^	MS	
Quercetin-3′-*O*-sulfate	99	15.354	0.70	248, 265, 366	380.99	
Quercetin-4′-*O*-sulfate	97	15.259	0.67	252, 264, 362	380.99	
Quercetin-3-*O*-sulfate	80	8.834	0,75	256, 351	380.99	
Quercetin di-*O*-sulfates	91	11.225	0.34	249, 265, 367	482.93	Na+
					498.90	K+

^a^ By HPLC; ^b^ By TLC, mobile phase ethylacetate/methanol/HCO_2_H 4:1:0.01; ^c^ Using PDA detector of HPLC chromatograph.

**Table 4:** Relative electronic energies (∆*E*, kcal.mol^−1^), relative Gibbs energies (∆*G*, kcal.mol^−1^), ionization potential (IP, eV) and O-H bond dissociation enthalpies (BDEs, kcal.mol^−1^) of quercetin derivatives.

Compound	∆*E* ^a^	∆*G* ^a^	IP	BDEs		
				3-OH	3′-OH	4′-OH
Quercetin	-	-	6.2	78.2	76.8	74.1
Quercetin-3′-*O*-sulfate	0.0	0.0	6.1	77.9	-	81.3
Quercetin-4′-*O*-sulfate	1.0	1.2	6.2	78.4	83.4	-
Quercetin-3′,4′-*O*-disulfate	8.5	8.4	6.1	78.1	-	-
Quercetin-7,3′-*O*-disulfate	0.0	0.0	6.1	77.5	-	81.1
Quercetin-7,4′-*O*-disulfate	0.9	0.6	6.2	79.0	83.2	-

^a^ Relative electronic and Gibbs energies were calculated for monosulfates or disulfates, with respect to the most stable mono- or disulfate regioisomer, respectively.

**Table 5:** Antioxidant capacity of quercetin sulfates in comparison with non-conjugated quercetin.

Compound	DPPH (IC_50_, μM) ^a^	ABTS^+^ (TE) ^b^	FCR (GAE) ^c^	DMPD (CE) ^d^	FRAP (FE) ^e^	ILP (IC_50_, μM) ^f^
Quercetin	3.41 ± 0.16	1.92 ± 0.08 ^h^	1.03 ± 0.08 ^i^	1.12 ± 0.09 ^j^	0.84 ± 0.05	19.8 ± 0.3
Quercetin-3′-*O*-sulfate	6.26 ± 0.86 ^g^	1.86 ± 0.08 ^h^	1.52 ± 0.09 ^h^	0.99 ± 0.04 ^j^	1.83 ± 0.07	34.3 ± 0.5
Quercetin-4′-*O*-sulfate	23.0 ± 1.1	1.90 ± 0.08 ^h^	1.01 ± 0.11 ^i^	1.14 ± 0.03 ^j^	2.27 ± 0.04	9.32 ± 0.12
Quercetin-di-*O-*sulfates	7.35 ± 0.17 ^g^	1.44 ± 0.13	1.72 ± 0.20 ^h^	1.60 ± 0.15	1.39 ± 0.02	48.3 ± 1.5

Data are presented as mean ± standard error from at least three independent measurements performed in triplicates. ^a^ 1,1-Diphenyl-2-picrylhydrazyl; ^b^ 2,2′-azinobis-(3-ethylbenzothiazoline-6-sulfonic acid) cation radical scavenging (trolox equivalents); ^c^ Folin-Ciocalteau reagent reduction (gallic acid equivalents); ^d^ *N*,*N-*dimethyl-*p*-phenylenediamine radical scavenging (vitamin C equivalents); ^e^ ferric reducing antioxidant power (Fe^2+^ equivalents); ^f^ inhibition of lipoperoxidation of rat liver microsomal membranes induced by *tert*-butylhydroperoxide; ^g–j^ The values marked with the same letter are not significantly different.

**Table 6:** Distance of center-of-mass (<z_COM_>), B-ring (<z_B-ring_>), C-rings (<z_C-ring_>), and 3-OH group (<z_3-OH_>) of quercetin sulfates to center of POPC lipid bilayer.

Compound	<z_COM_> (Å)	<z_B-ring_> (Å)	<z_C-ring_> (Å)	<z_3-OH_> (Å)
Quercetin	12.5 ± 2.0	14.1 ± 1.9	12.1 ± 2.1	12.1 ± 2.2
Quercetin-3′-*O*-sulfate	14.0 ± 2.1	15.4 ± 2.3	12.5 ± 2.1	12.6 ± 2.4
Quercetin-4′-*O*-sulfate	13.4 ± 1.8	14.3 ± 1.9	11.8 ± 1.9	10.8 ± 2.0
Quercetin-3′,4′-di-*O*-sulfate	16.5 ± 1.7	17.4 ± 1.7	13.9 ± 1.7	14.2 ± 1.9
Quercetin-7,3′-di-*O*-sulfate	14.8 ± 1.8	14.8 ± 2.1	13.9 ± 1.9	13.0 ± 2.1
Quercetin-7,4′-di-*O*-sulfate	14.4 ± 1.7	14.4 ± 1.8	12.9 ± 1.7	11.3 ± 1.8

## Supplementary Materials

Supplementary materials can be found at www.mdpi.com/1422-0067/18/11/2231/s1.

## Abbreviations

ABTS	2,2′-Azinobis-(3-ethylbenzothiazoline-6-sulfonic acid)
PME	Particle-Mesh Ewald
AST	Arylsulfotransferase
FF	Force field
CE	Vitamin C equivalents
COSY	Correlation spectroscopy
DFT	Density functional theory
DMPD	*N*,*N-*Dimethyl-*p*-phenylenediamine
DMSO	Dimethyl sulfoxide
DPPH	1,1-Diphenyl-2-picrylhydrazyl
FCR	Folin-Ciocalteau reduction
FRAP	Ferric reducing antioxidant power
GAE	Gallic acid equivalents
GAFF	Generalized amber force field
HMBC	Heteronuclear multiple-bond correlation spectroscopy
HSQC	Heteronuclear single-quantum correlation spectroscopy
IC_50_	The concentration of the tested compound that inhibited the reaction by 50%
IPs	Ionization potentials
Lpx	Lipid peroxidation
MD	Molecular dynamics
PBS	Phosphate-buffered saline
*p*-NP	*p*-Nitrophenol
*p*-NPS	*p*-Nitrophenylsulfate
POPC	1-Palmitoyl-2-oleoyl-*sn*-glycero-3-phosphocholine
RESP	Restrained fit of electrostatic potential
TBARS	Thiobarbituric acid reactive substances
TE	Trolox-equivalents
TOCSY	Two-dimensional nuclear magnetic resonance spectroscopy
