# Supplementary material for: Chemoenzymatic Preparation and Biophysical Properties of Sulfated Quercetin Metabolites
Source: Int J Mol Sci. 2017 Oct 25;18(11):2231. doi: 10.3390/ijms18112231 (PMC5713201; doi:10.3390/ijms18112231)
Supplement: Supplementary file 1 [file ijms-18-02231-s001.pdf]

Chemoenzymatic Preparation and Biophysical Properties of Sulfated Quercetin Metabolites

Kateřina Valentová[1,*], Kristýna Káňová[1], Florent Di Meo[2,*], Helena Pelantová[1], Christopher Steven Chambers[1], Lenka Rydlová[1], Lucie Petrásková[1], Alena Křenková[1], Josef Cvačka[3], Patrick Trouillas[2,4], Vladimír Křen[1]

[1] Institute of Microbiology, Czech Academy of Sciences, Vídeňská 1083, CZ-142 20 Prague, Czech Republic; astriik@gmail.com (K.K.); pelantova@biomed.cas.cz (H.P.); christopher.chambers@biomed.cas.cz (C.S.C.); petraskova@biomed.cas.cz (L.P.); alenka.petrickova@gmail.com (A.K.); kren@biomed.cas.cz (V.K.).

[2]INSERM U850, Univ. Limoges, School of Pharmacy, 2 rue du Docteur Marcland, 87025 Limoges, France; patrick.trouillas@unilim.fr (P.T.).

[3]Institute of Organic Chemistry and Biochemistry, Czech Academy of Sciences, Flemingovo nám. 2, CZ 16610 Prague, Czech Republic; cvacka@uochb.cas.cz.

[4]Regional Centre of Advanced Technologies and Materials, Department of Physical Chemistry, Faculty of Science, Palacký University, tř. 17. listopadu 12, 771 46 Olomouc, Czech Republic

Table of Content:

Figure S1. HPLC chromatograms of quercetin sulfation reaction mixture at (a) 1 h, (b) 5 h, (c) 24 h. 2
Figure S2. Distance of quercetin derivatives to lipid bilayer center over MD simulations. 3
Table S1. Average non-covalent interactions between solute and (a) lipids or (b) lipid tails. 3
Figure S3. ^{1}H NMR spectrum of quercetin-3'-*O*-sulfate (600.23 MHz, DMSO-d_6, 30 °C). 4
Figure S4. ^{13}C NMR spectrum of quercetin-3'-*O*-sulfate (150.93 MHz, DMSO-d_6, 30 °C). 4
Figure S5. Mass spectrum (ESI−) of quercetin-3'-O-sulfate. 5
Figure S6. HPLC chromatogram of quercetin-3'-*O*-sulfate. 5
Figure S7. ^{1}H NMR spectrum of quercetin-4'-*O*-sulfate (600.23 MHz, DMSO-d_6, 30 °C). 6
Figure S8. ^{13}C NMR spectrum of quercetin-4'-*O*-sulfate (150.93 MHz, DMSO-d_6, 30 °C). 6
Figure S9. Mass spectrum (ESI−) of quercetin-4'-O-sulfate. 7
Figure S10. HPLC chromatogram of quercetin-4'-*O*-sulfate. 7
Figure S11. ^{1}H NMR spectrum of quercetin-3-*O*-sulfate (399.87 MHz, DMSO-d_6, 30 °C). 8
Figure S12. ^{13}C NMR spectrum of quercetin-3-*O*-sulfate (100.55 MHz, DMSO-d_6, 30 °C). 8
Figure S13. Mass spectrum (ESI−) of quercetin-3-O-sulfate. 9
Figure S14. HPLC chromatogram of quercetin-3-*O*-sulfate. 9
Figure S15. Expanded ^{1}H NMR spectrum of quercetin-di-*O*-sulfates mixture (600.23 MHz, DMSO-d_6, 30 °C)...... 10
Figure S16. Expanded ^{13}C NMR spectrum of quercetin-di-*O*-sulfates mixture (150.93 MHz, DMSO-d_6, 30 °C). 10
Figure S17. Mass spectrum (ESI−) of quercetin-di-O-sulfates. 11
Figure S18. HPLC chromatogram of quercetin-di-*O*-sulfates mixture.. 11

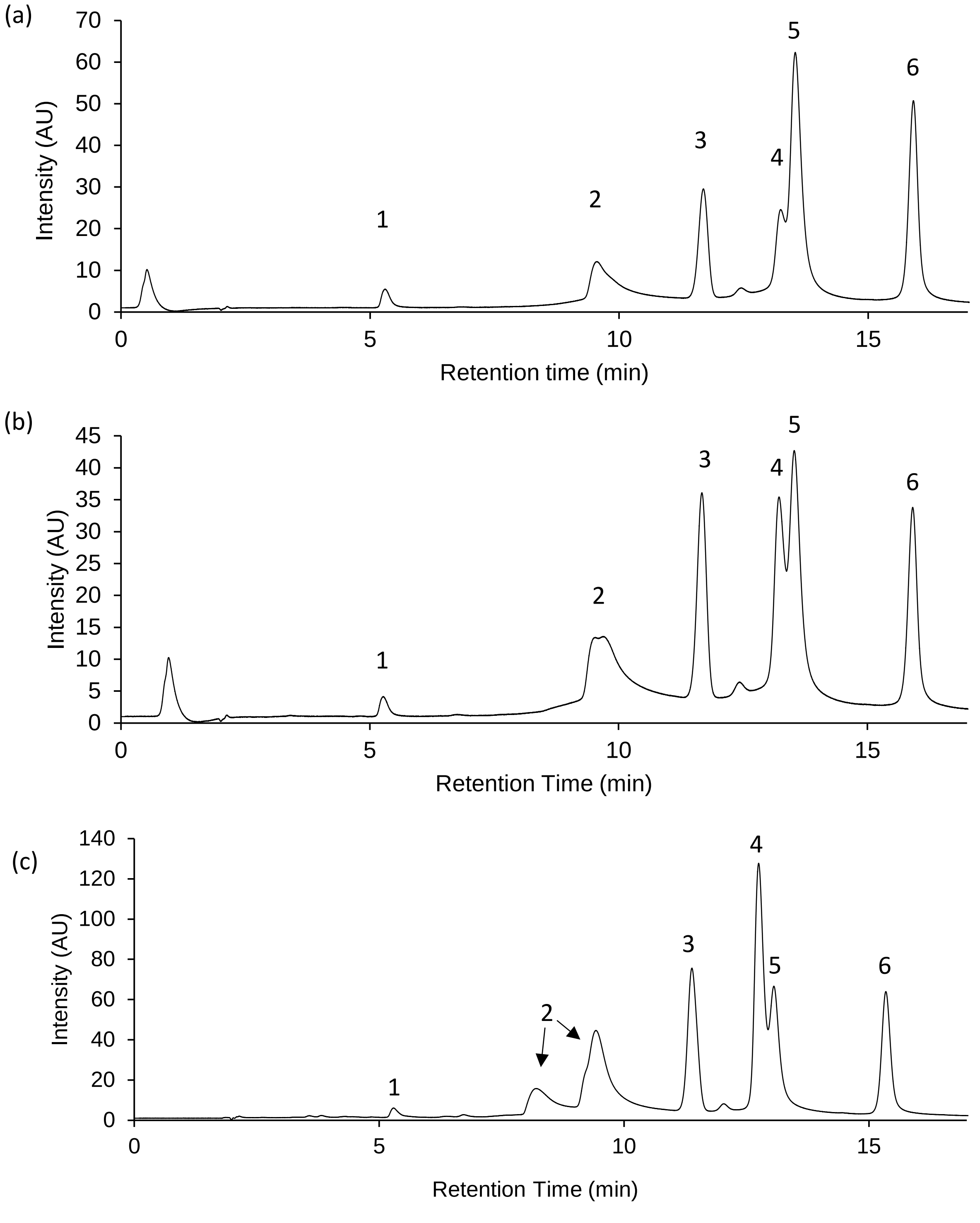

Figure S1. HPLC chromatograms of quercetin sulfation reaction mixture at (a) 1 h, (b) 5 h, (c) 24 h. Peak assignment: 1 – *p*-NP, 2 – quercetin disulfates, 3 – *p*-NPS, 4 – quercetin-3′-*O*-sulfate, 5 - quercetin-4′-*O*-sulfate, 6 – quercetin; detection at 370 nm.

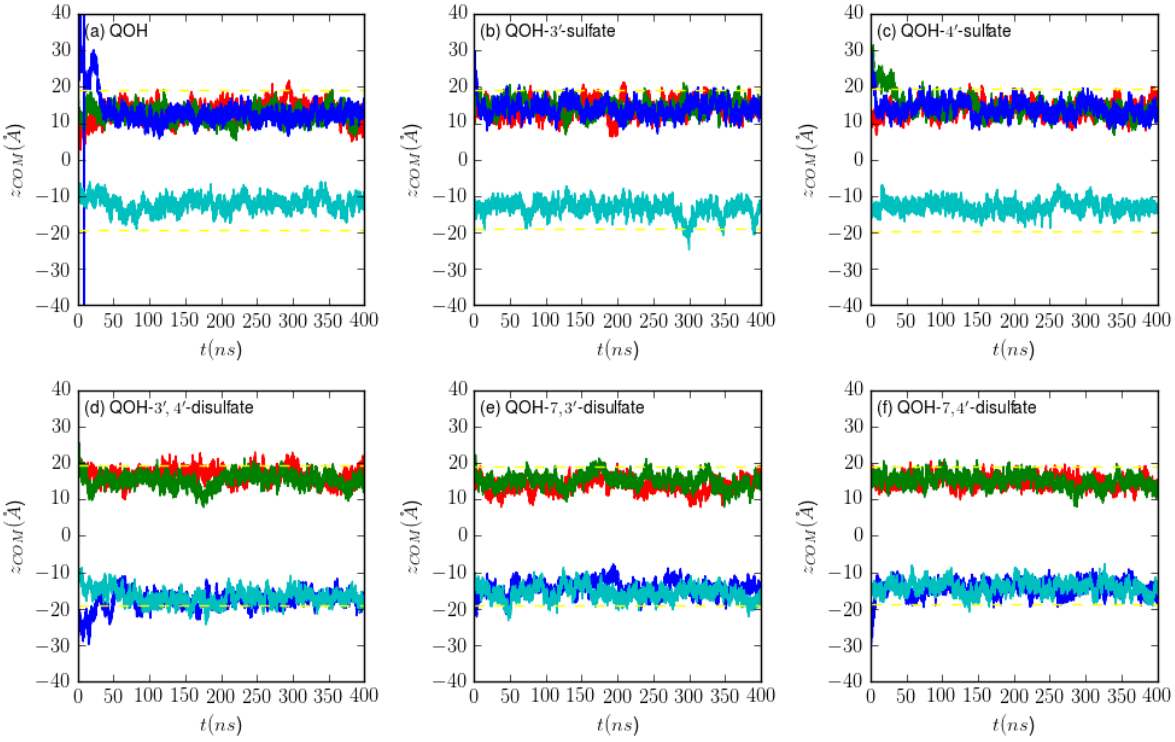

Figure S2. Distance of quercetin derivatives to lipid bilayer center over MD simulations. Each quercetin replica is depicted in red, green blue and cyan. Average phosphate distances to lipid center is plotted in yellow.

Table S1. Average non-covalent interactions between solute and (a) lipids or (b) lipid tails.

(a)

	E_{int}	E_{elec}	E_{vdw}
quercetin	-78.0±16.2	-47.7±16.4	-30.3±7.7
quercetin 3´-O-sulfate	-134.7±27.2	-92.6±22.7	-42.2±8.7
quercetin 4´-O-sulfate	-138.6±20.2	-94.1±17.2	-44.5±8.0
quercetin 3',4´-O-disulfate	-288.7±53.8	-245.5±47.4	-43.2±10.4
quercetin-7',3´-O-disulfate	-222.2±31.7	-170.1±28.4	-52.1±7.5
quercetin-7',4´-O-disulfate	-216.1±30.2	-163.1±26.0	-53.0±8.5

(b)

	E_{int}	E_{elec}	E_{vdw}
quercetin	-24.1±7.0	-0.9±0.6	-23.3±6.8
quercetin 3´-O-sulfate	-23.0±6.7	-0.4±1.2	-22.6±6.2
quercetin 4´-O-sulfate	-29.4±7.3	-1.2±	-28.2±6.6
quercetin 3',4´-O-disulfate	-17.3±6.4	0.2±	-17.6±6.1
quercetin-7',3´-O-disulfate	-25.6±5.9	-1.2±	-24.4±4.9
quercetin-7',4´-O-disulfate	-30.5±6.8	-1.4±	-29.1±5.9

total (E_{int}, kcal.mol^{-1}), electrostatic (E_{elec}, kcal.mol^{-1}) and van der Waals energies (E_{vdw}, kcal.mol^{-1}) over the last 200 ns

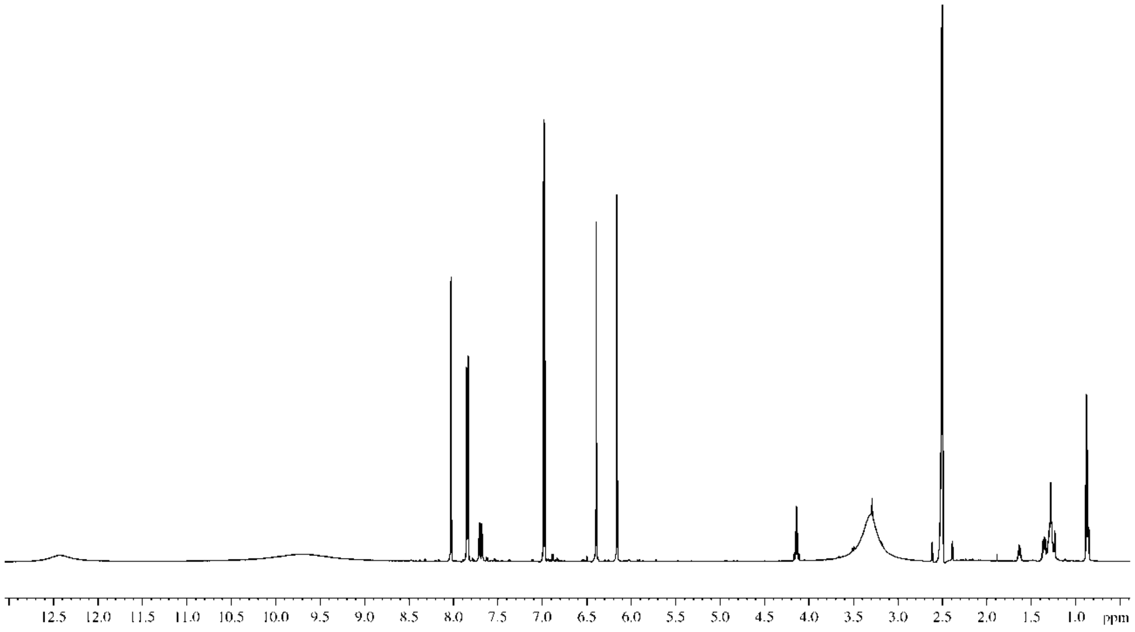

Figure S3. ^{1}H NMR spectrum of quercetin-3′-*O*-sulfate (600.23 MHz, DMSO-d_6, 30 °C).

Signal assignment: 6.158 (1H, d, J = 2.0 Hz, H-6), 6.392 (1H, d, J = 2.0 Hz, H-8), 6.977 (1H, d, J = 8.6 Hz, H-5′), 7.841 (1H, dd, J = 8.6, 2.3 Hz, H-6′), 8.026 (1H, d, J = 2.3 Hz, H-2′)

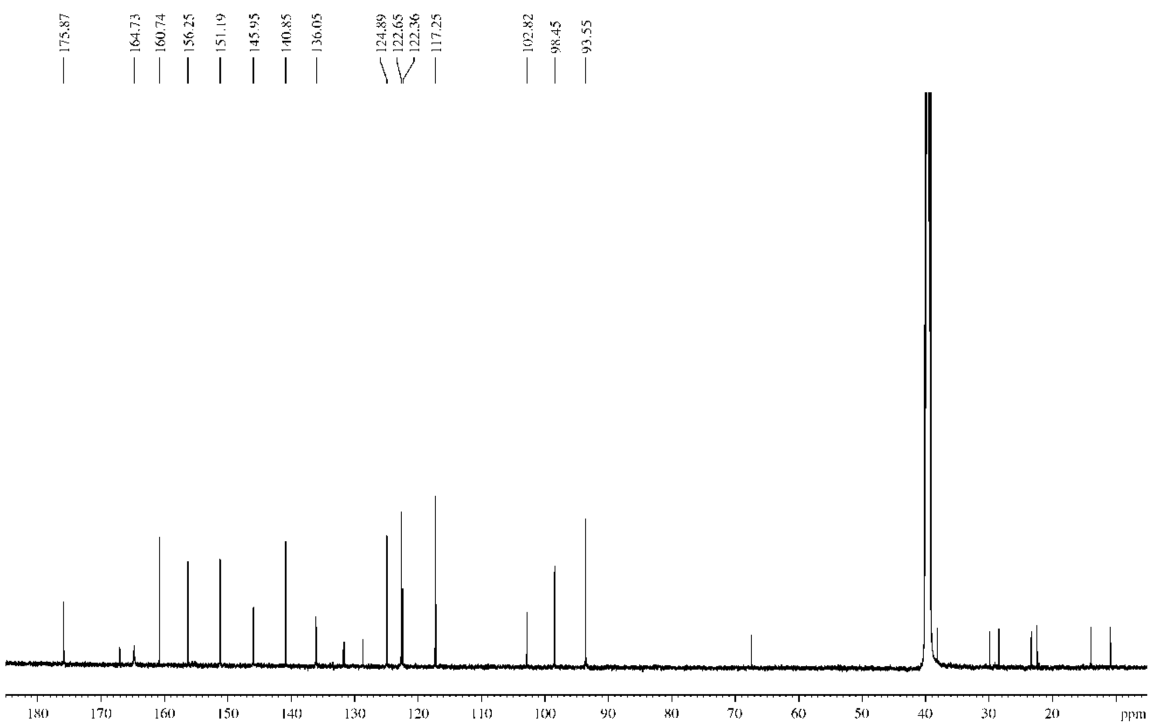

Figure S4. ^{13}C NMR spectrum of quercetin-3′-*O*-sulfate (150.93 MHz, DMSO-d_6, 30 °C).

Signal assignment: 93.55 (C-8), 98.45 (C-6), 102.82 (C-10), 117.25 (C-5′), 122.36 (C-1′), 122.65 (C-2′), 124.89 (C-6′), 136.05 (C-3), 140.85 (C-3′), 145.95 (C-2), 151.19 (C-4′), 156.25 (C-9), 160.74 (C-5), 164.73 (C-7), 175.87 (C-4)

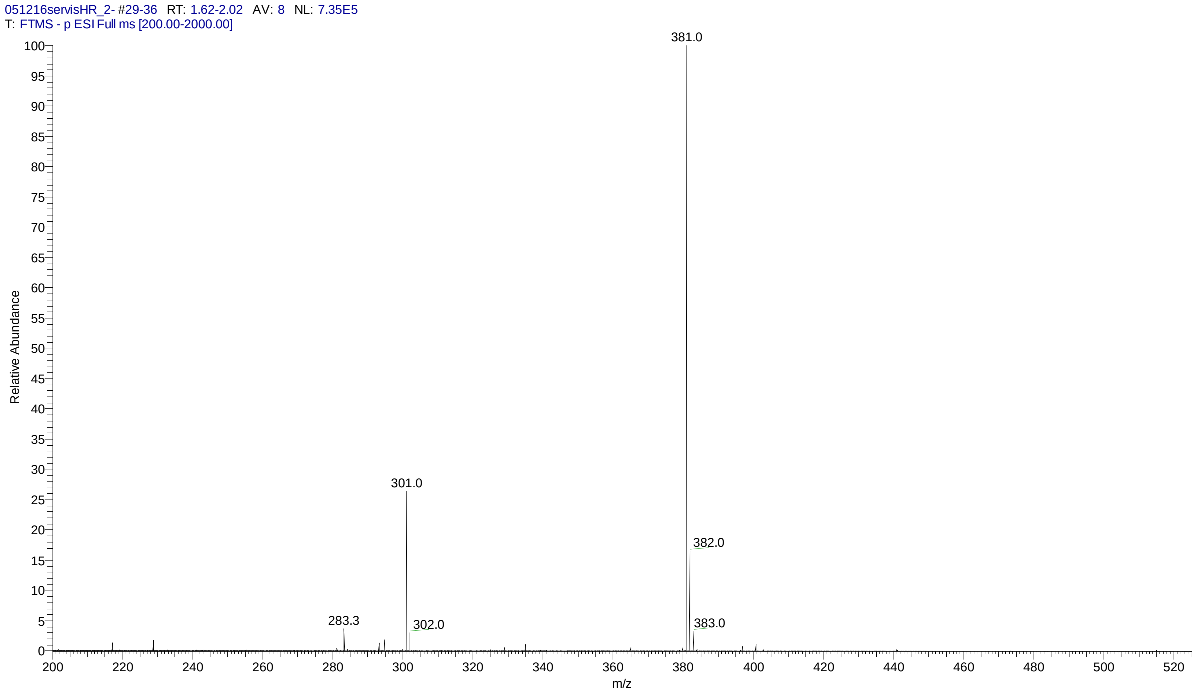

Figure S5. Mass spectrum (ESI−) of quercetin-3′-O-sulfate.

$[M-H]^-$, *m/z* 381.0; $[M-H-SO_3]^-$, *m/z* 301.0.

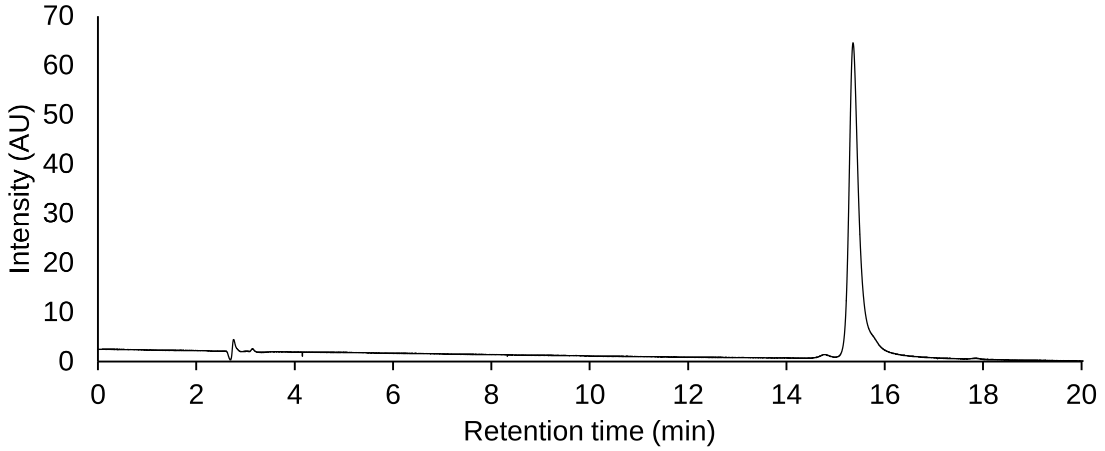

Figure S6. HPLC chromatogram of quercetin-3′-*O*-sulfate.

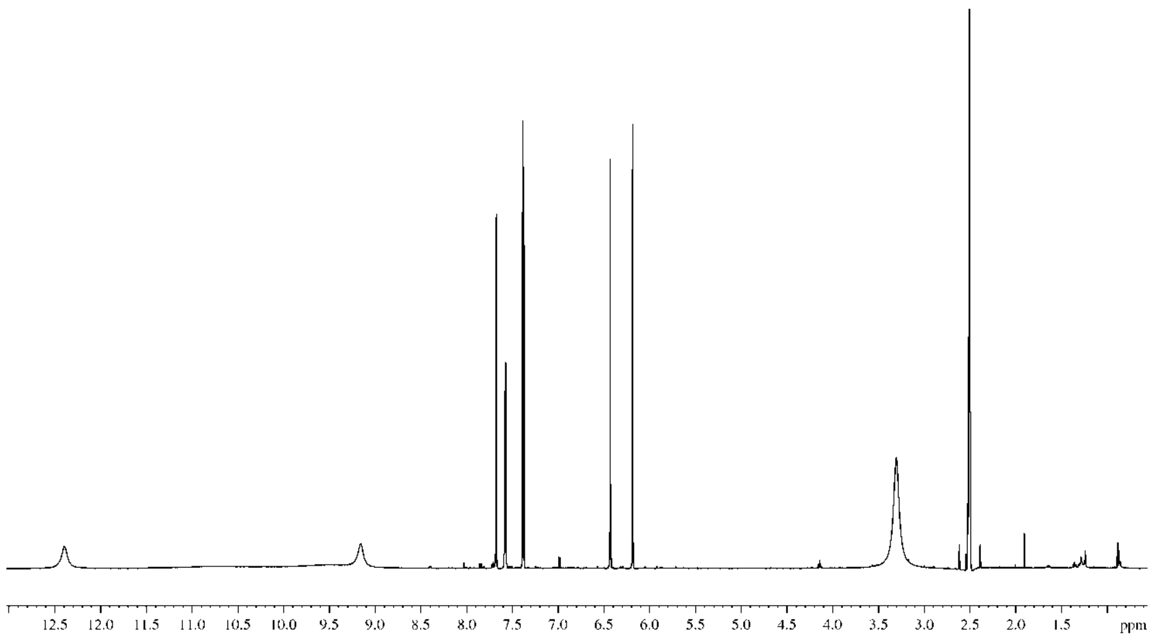

Figure S7. ^{1}H NMR spectrum of quercetin-4′-*O*-sulfate (600.23 MHz, DMSO-d_6, 30 °C).

Signal assignment: 6.181 (1H, d, *J* = 2.1 Hz, H-6), 6.428 (1H, d, *J* = 2.1 Hz, H-8), 7.377 (1H, d, *J* = 8.6 Hz, H-5′), 7.573 (1H, dd, *J* = 8.6, 2.2 Hz, H-6′), 7.672 (1H, d, *J* = 2.2 Hz, H-2′)

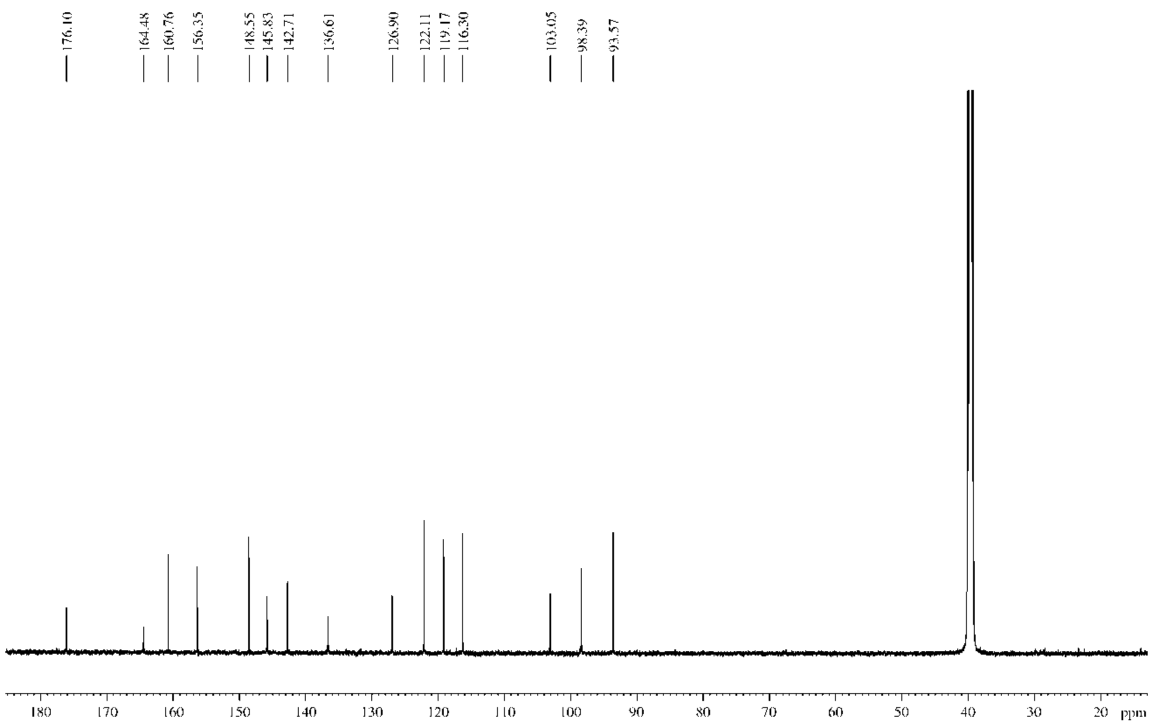

Figure S8. ^{13}C NMR spectrum of quercetin-4′-*O*-sulfate (150.93 MHz, DMSO-d_6, 30 °C).

signal assignment: 93.57 (C-8), 98.39 (C-6), 103.05 (C-10), 116.30 (C-2′), 119.17 (C-6′), 122.11 (C-5′), 126.90 (C-1′), 136.61 (C-3), 142.71 (C-4′), 145.83 (C-2), 148.55 (C-3′), 156.35 (C-9), 160.76 (C-5), 164.48 (C-7), 176.10 (C-4)

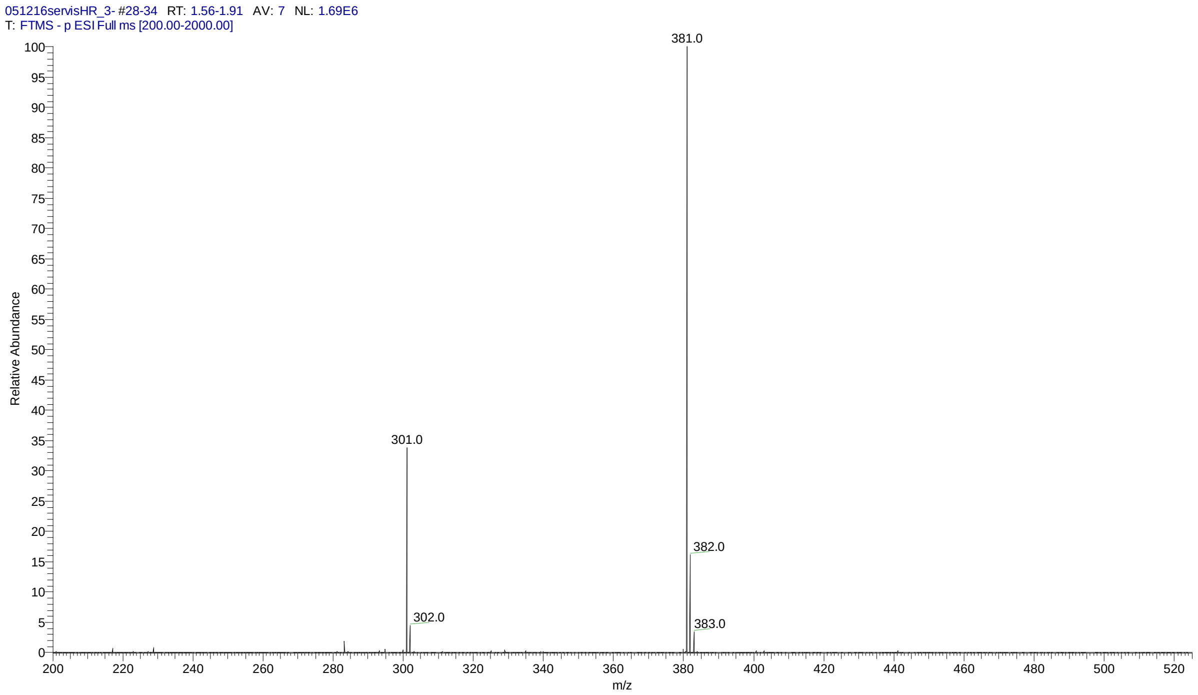

Figure S9. Mass spectrum (ESI−) of quercetin-4′-O-sulfate.

[M – H]$^-$, *m/z* 381.0; [M – H – SO_3]$^-$, *m/z* 301.0.

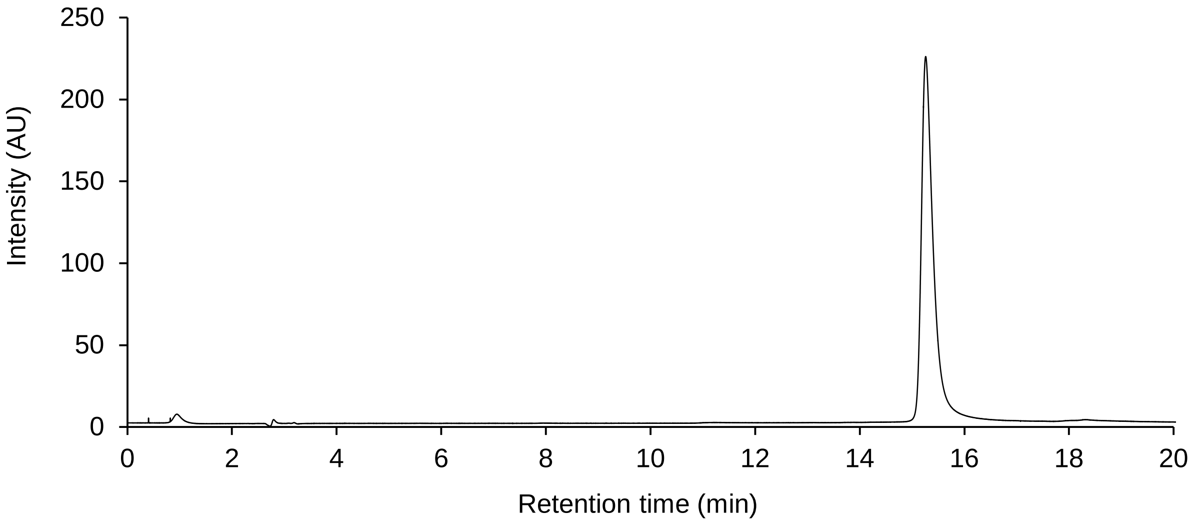

Figure S10. HPLC chromatogram of quercetin-4′-*O*-sulfate.

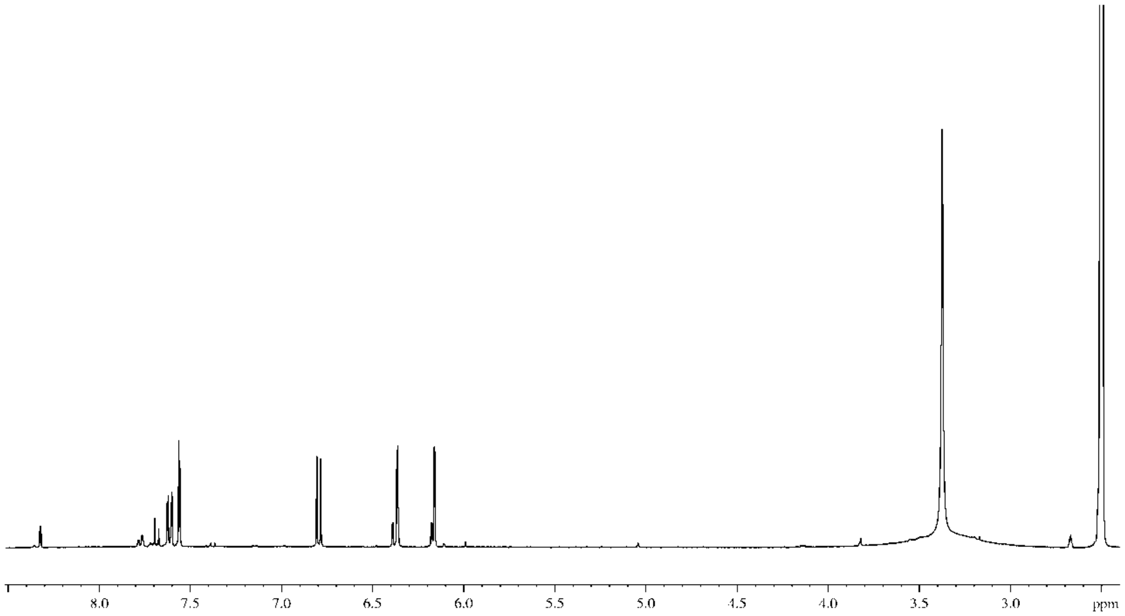

Figure S11. ^{1}H NMR spectrum of quercetin-3-*O*-sulfate (399.87 MHz, DMSO-d_6, 30 °C).

signal assignment: ^{1}H NMR: 6.159 (1H, d, *J* = 2.1 Hz, H-6), 6.363 (1H, d, *J* = 2.1 Hz, H-8), 6.795 (1H, d, *J* = 8.5 Hz, H-5′), 7.560 (1H, d, *J* = 2.2 Hz, H-2′), 7.613 (1H, dd, *J* = 8.5, 2.2 Hz, H-6′)

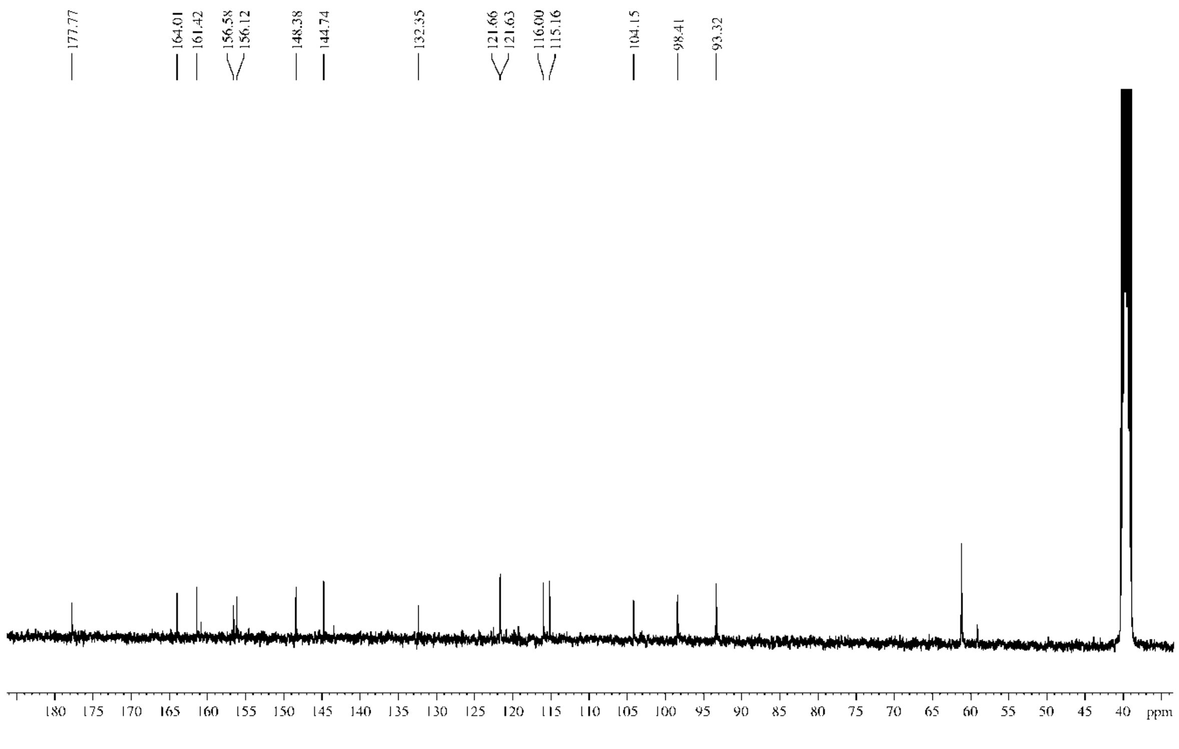

Figure S12. ^{13}C NMR spectrum of quercetin-3-*O*-sulfate (100.55 MHz, DMSO-d_6, 30 °C).

signal assignment: 93.32 (C-8), 98.41 (C-6), 104,15 (C-10), 115.16 (C-5′), 116.00 (C-2′), 121.63[a] (C-1′), 121.66[a] (C-6′), 132.35 (C-3), 144.74 (C-3′), 148.38 (C-4′), 156.12 (C-9), 156.58 (C-2), 161.42 (C-5), 164.01 (C-7), 177.77 (C-4) [a] … might be interchanged

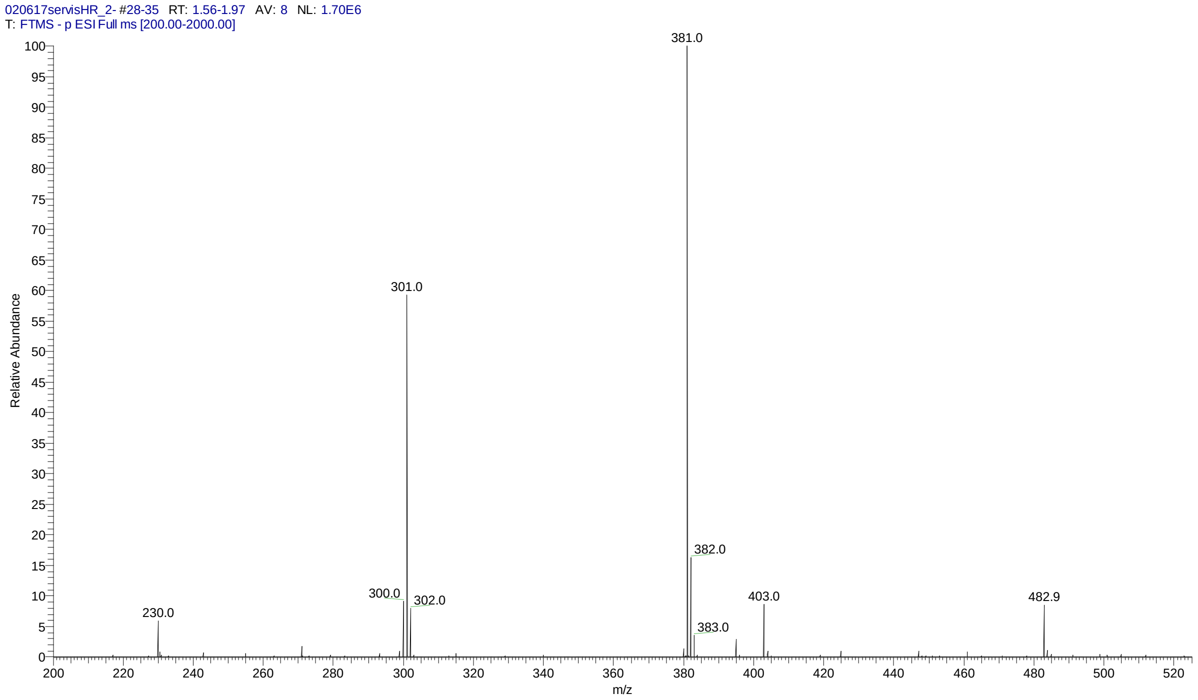

Figure S13. Mass spectrum (ESI−) of quercetin-3-O-sulfate.

$[M-H]^-$, *m/z* 381.0; $[M-H-SO_3]^-$, *m/z* 301.0. Signals of a disulfate were found at *m/z* 482.9 ($[M-2H+Na]^-$), *m/z* 403.0 ($[M-2H+Na-SO_3]^-$), and *m/z* 230.0 ($[M-2H]^{2-}$).

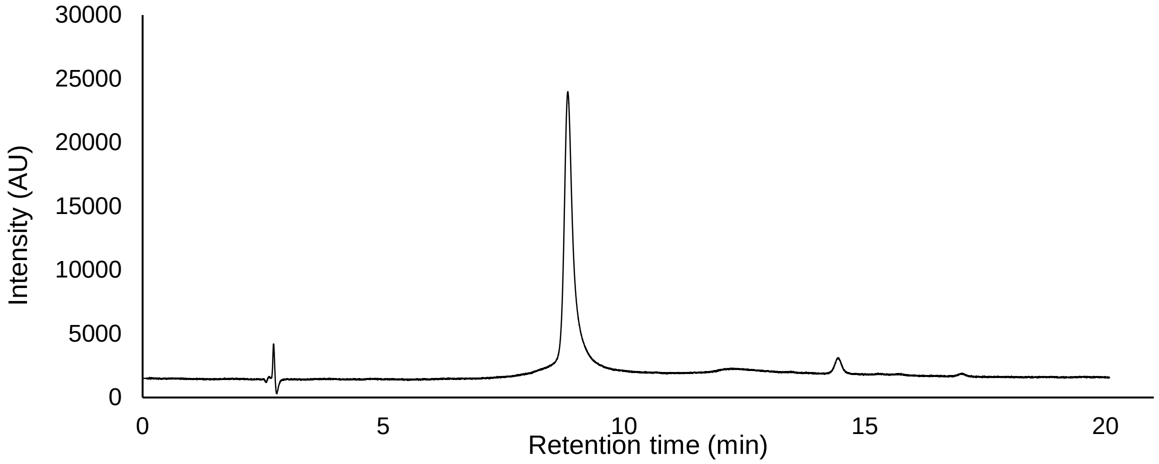

Figure S14. HPLC chromatogram of quercetin-3-*O*-sulfate.

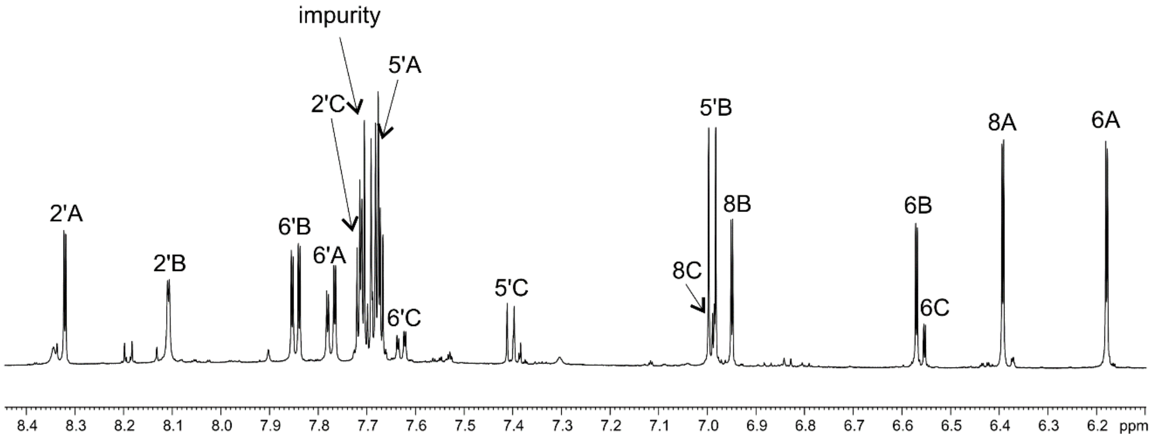

Figure S15. Expanded ^{1}H NMR spectrum of quercetin-di-*O*-sulfates mixture (600.23 MHz, DMSO-d_6, 30 °C). Signals of individual components are marked as **A**: quercetin-3',4'-di-*O*-sulfate, **B**: quercetin-7,3'-di-*O*-sulfate, and **C**: quercetin-7,4'-di-*O*-sulfate.

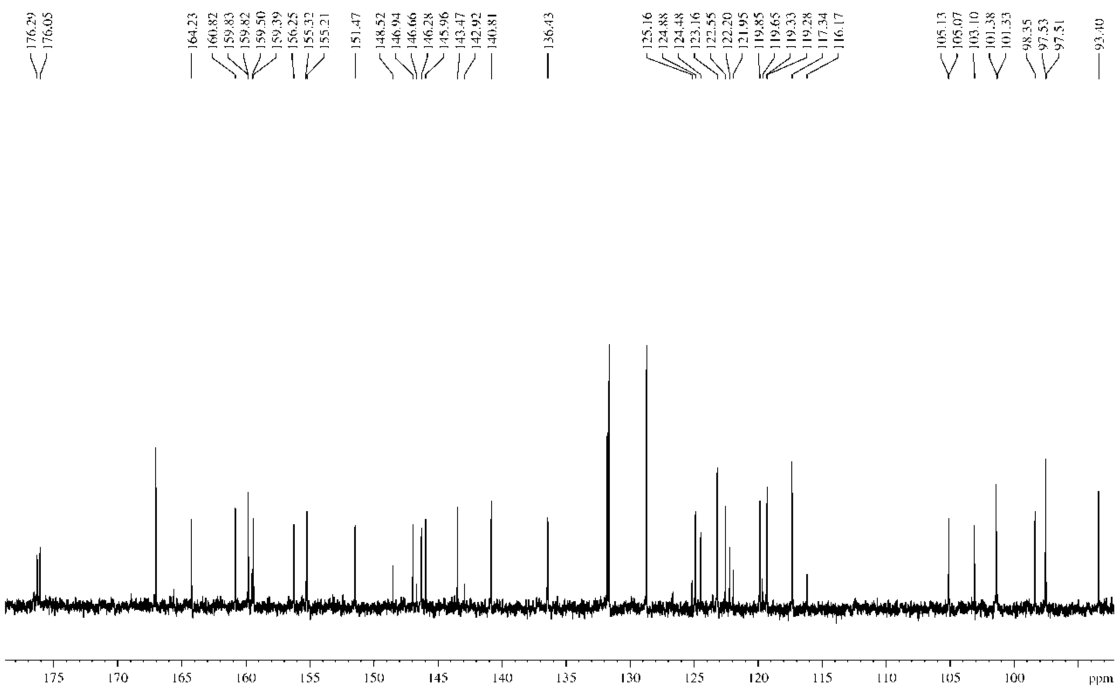

Figure S16. Expanded ^{13}C NMR spectrum of quercetin-di-*O*-sulfate mixture (150.93 MHz, DMSO-d_6, 30 °C).

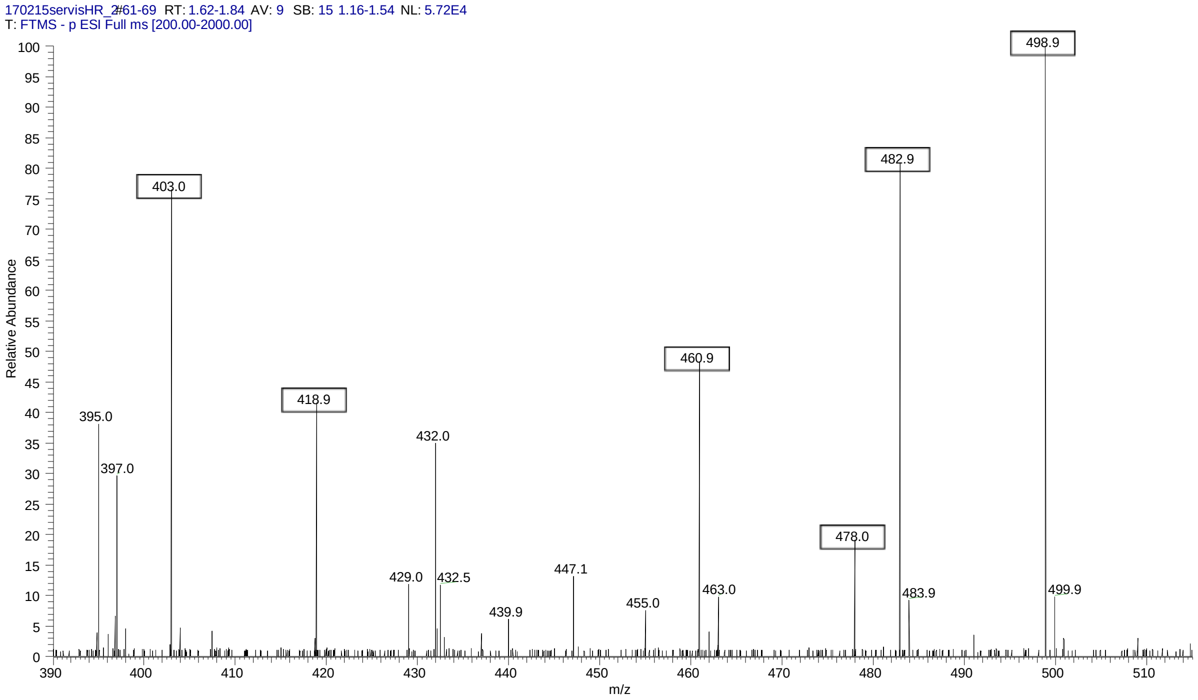

Figure S17. Mass spectrum (ESI−) of quercetin-di-O-sulfates.

$[M - 2H + K]^-$, m/z 498.9; $[M - 2H + Na]^-$, m/z 482.9; $[M - 2H + NH_4]^-$, m/z 478.0; $[M - H]^-$, m/z 460.9; $[M - 2H + K - SO_3]^-$, m/z 418.9; $[M - 2H + Na - SO_3]^-$, m/z 403.0.

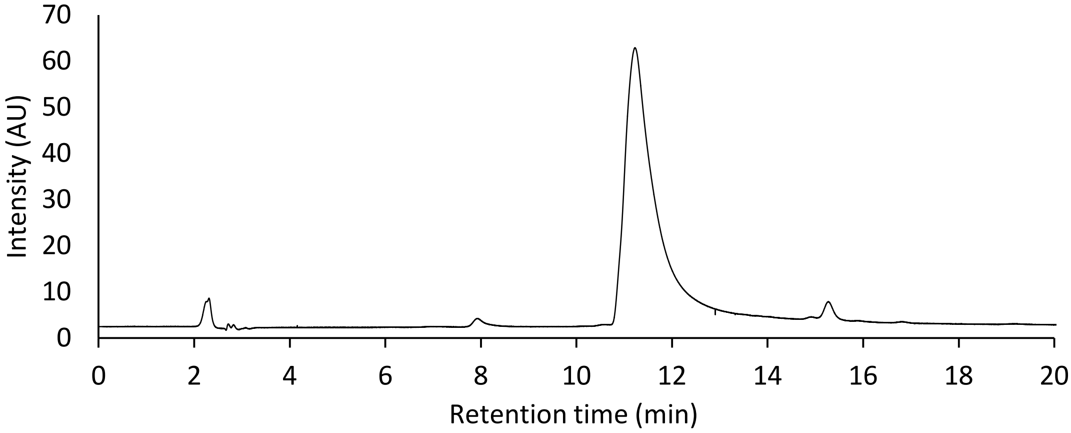

Figure S18. HPLC chromatogram of quercetin-di-*O*-sulfate mixture.
